# Next-generation mapping of the salicylic acid signaling hub and transcriptional cascade

**DOI:** 10.1016/j.molp.2024.08.008

**Published:** 2024-08-22

**Authors:** Jordan Powers, Xing Zhang, Andres V. Reyes, Raul Zavaliev, Roni Ochakovski, Shou-Ling Xu, Xinnian Dong

**Affiliations:** 1 Howard Hughes Medical Institute, Duke University, Durham, NC 27708, USA; 2 University Program in Genetics and Genomics, Duke University, Durham, NC 27708, USA; 3 Carnegie Institute for Science, Stanford University, Stanford, CA 94305, USA; 4 Present address: Department of Biology, Brookhaven National Laboratory, Upton, NY 11973, USA

**Keywords:** salicylic acid-induced transcription, NPR1, TGA, WRKY TFs, greenCUT&RUN, TurboID

## Abstract

For over 60 years, salicylic acid (SA) has been known as a plant immune signal required for basal and systemic acquired resistance. SA activates these immune responses by reprogramming ~20% of the transcriptome through NPR1. However, components in the NPR1 signaling hub, which appears as nuclear condensates, and the NPR1 signaling cascade have remained elusive due to difficulties in studying this transcriptional cofactor, whose chromatin association is indirect and likely transient. To overcome this challenge, we applied TurboID to divulge the NPR1 proxiome, which detected almost all known NPR1 interactors as well as new components of transcription-related complexes. Testing of new components showed that chromatin remodeling and histone demethylation contribute to SA-induced resistance. Globally, the NPR1 proxiome has a striking similarity to the proxiome of GBPL3 that is involved in SA synthesis, except for associated transcription factors (TFs), suggesting that common regulatory modules are recruited to reprogram specific transcriptomes by transcriptional cofactors, like NPR1, through binding to unique TFs. Stepwise green fluorescent protein-tagged factor cleavage under target and release using nuclease (greenCUT&RUN) analyses showed that, upon SA induction, NPR1 initiates the transcriptional cascade primarily through association with TGACG-binding TFs to induce expression of secondary TFs, predominantly WRKYs. Further, WRKY54 and WRKY70 were identified to play a major role in inducing immune-output genes without interacting with NPR1 at the chromatin. Moreover, loss of condensate formation function of NPR1 decreases its chromatin association and transcriptional activity, indicating the importance of condensates in organizing the NPR1 signaling hub and initiating the transcriptional cascade. Collectively, this study demonstrates how combinatorial applications of TurboID and stepwise greenCUT&RUN transcend traditional genetic methods to globally map signaling hubs and transcriptional cascades for in-depth explorations.

## INTRODUCTION

In plants, a local infection can often lead to systemic acquired resistance through the accumulation of the phytohormone salicylic acid (SA) (Malamy et al., 1990; Metraux et al., 1990) which, in *Arabidopsis thaliana*, results in changes of up to 20% of its transcriptome (Wang et al., 2006). This process is mediated by the downstream signaling component nonexpresser of PR genes 1 (NPR1); mutating it leads to a drastic loss of the transcriptional response and enhanced susceptibility to primary and secondary infection (Cao et al., 1994). Further studies have identified NPR1, and its homologs NPR1-like protein 3 and 4 (NPR3/4), as SA receptors in *Arabidopsis* with different binding affinities (Fu et al., 2012; Wu et al., 2012; Ding et al., 2018; Wang et al., 2020; Kumar et al., 2022). Because the NPR1 protein lacks a DNA-binding domain, it has been proposed to function as a transcriptional cofactor for transcription factors (TFs), such as TGACG-binding (TGAs) (Zhang et al., 1999; Despres et al., 2000; Zhou et al., 2000) and WRKYs (Saleh et al., 2015; Chen et al., 2019). However, our knowledge of how NPR1 functions molecularly to orchestrate the transcriptome-wide changes in response to SA is still limited by the insufficient sensitivity of current methodologies for investigating a transcriptional cofactor like NPR1. A recent structural study of the NPR1 complex with the TGA3 TF showed that NPR1 serves its transcriptional coactivator role as a dimer by bridging two dimeric TGA3 molecules; i.e., (TGA3)_2_-(NPR1)_2_-(TGA3)_2_ (Kumar et al., 2022). The presence of (NPR1)_2_-(TGA3)_2_ intermediates in the cryoelectron microscopy samples suggests that the NPR1 dimer may function as a platform to nucleate TFs in an enhanceosome. This raises the following question: does NPR1 interact with different TFs concurrently in response to SA to activate the myriad of output genes or, alternatively, initiate the reprogramming through a transcriptional cascade? Besides TFs, NPR1 is likely to be associated with large molecular complexes in response to SA because of the nuclear and cytoplasmic condensates detected for the protein (Saleh et al., 2015; Zavaliev et al., 2020). While the SA-induced NPR1 cytoplasmic condensates (cSINCs) have been characterized (Zavaliev et al., 2020), the contents and function of SA-induced NPR1 nuclear condensates remain elusive. Therefore, a comprehensive study of NPR1’s proximal partners in the nucleus and a stepwise dissection of NPR1 transcriptional targets are essential for elucidating the molecular mechanisms by which this master immune regulator reprograms the transcriptome.

## RESULTS

### Label-free quantitative analysis of the NPR1 proxiome using TurboID identifies core components that regulate gene expression and contribute to SA-induced resistance

**Figure 1.**
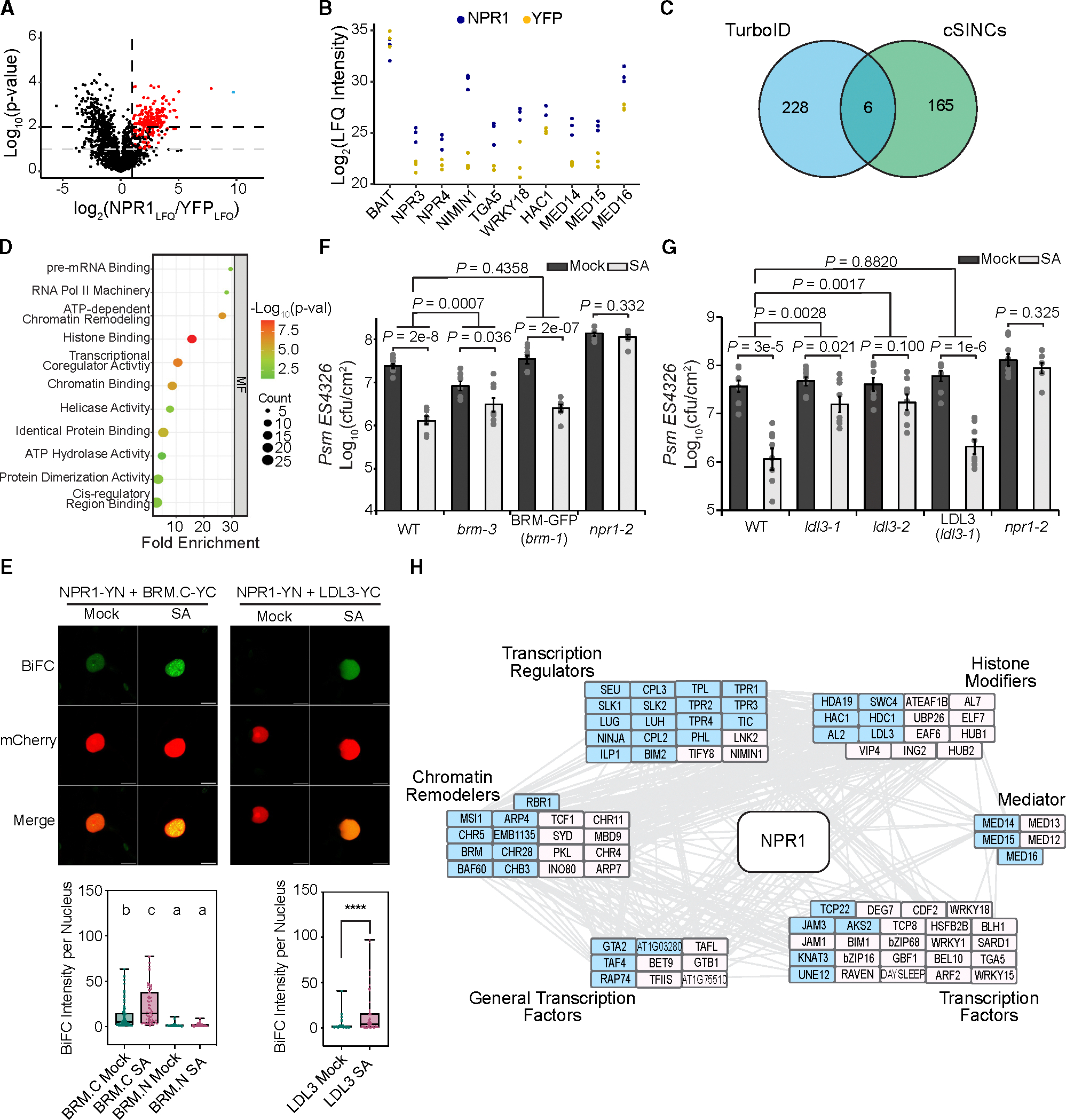
The NPR1 proxiome contains transcriptional machineries and chromatin remodelers shared by the GBPL3-proxiome. **(A)** Volcano plot of NPR1 proximal proteins 4 h after SA treatment, detected through TurboID biotin affinity purification followed by label-free quantification (LFQ) MS processed under mild conditions (Methods). Red points represent proteins that have an NPR1_LFQ_/YFP_LFQ_ ≥ 2 and *p* < 0.1 in both mild and harsh washing conditions (Methods) or *p* < 0.01 in at least one washing condition. The single blue point (on the right) represents NPR1. **(B)** Log_2_(maximum LFQ intensity) of NPR1–3 × HA-TbID, YFP-YFP-TbID (BAIT), and known NPR1 interactors in NPR1–3 × HA-TbID (NPR1) vs. YFP-YFP-TbID (YFP) samples. **(C)** Venn diagram comparing NPR1 proximal proteins identified in the current TurboID experiment with those identified in the cytoplasmic SA-induced NPR1 condensates (cSINCs) (Zavaliev et al., 2020). **(D)** Enriched molecular functions (MFs) of the 234 NPR1 proximal proteins. **(E)** Proximity between NPR1 and LDL3 or BRM. N-terminal YFP-fused NPR1 (NPR1-YN) and the C-terminal YFP-fused BRM C terminus (amino acids 953–2193) (BRM.C-YC) or cYFP-fused LDL3 (LDL3-YC) were co-expressed with the nuclear marker protein histone 2B fused to mCherry (HTB1-mCherry) in *N. benthamiana*. Plants were imaged after treatment with water (mock) or 1 mM SA for 8 h, and the BiFC intensities were measured from multiple nuclei; values were plotted on a box-and-whisker plot. Different letters indicate statistical significance based on an ordinary one-way ANOVA with Tukey’s multiple-comparisons tests (a single pooled variance). Asterisks indicate statistical significance tested by two-tailed unpaired Student’s *t*-test (****p < 0.0001). Scale bar, 10 μm. **(F and G)** WT, *npr1–2*, *brm-3, BRM:BRM-GFP/brm-1* **(F)**, *ldl3–1*, *ldl3–2*, and *LDL3-FLAG/ldl3–1* **(G)** treated with H_2_O (mock) or 1 mM SA for 24 h prior to inoculation with *Psm* ES4326 at OD_600_ = 0.001. Bacterial colony-forming units (CFUs) were measured 3 days post inoculation (*n* = 8; error bars represent SEM; two-sided *t*-test and two-way ANOVA were used for comparisons within and between genotypes, respectively). **(H)** Search Tool for the Retrival of Interacting Genes/Proteins (STRING) network analysis (Szklarczyk et al., 2019) of NPR1 proximal proteins relating to chromatin remodeling and transcriptional regulation. Blue shade, proteins shared with the GBPL3 proxiome (Tang et al., 2022).

To address the question of how the transcriptional reprogramming occurs after the SA-bound NPR1 dimer bridges the TGA TF complexes (Kumar et al., 2022), we generated stable transgenic plants expressing NPR1–3× hemagglutinin (HA) fused with a promiscuous biotin ligase, TurboID (Mair et al., 2019; Xu et al., 2023). The activity of the resulting NPR1–3 × HA-TurboID (NPR1-TbID) was validated by its ability to restore, in the *npr1–2* background, the induction of *PR1*, a known NPR1 target (Supplemental Figure 1A). We treated this transgenic line and the control (yellow fluorescent protein [YFP]-YFP-TurboID) with 1 mM SA followed by sample collection at 4 h, when NPR1 displays nuclear accumulation (Kinkema et al., 2000; Zavaliev et al., 2020) and the *PR1* gene expression pattern shows the most rapid increase (Saleh et al., 2015). Using label-free quantification of the liquid chromatography-tandem mass spectrometry (LC-MS/MS) method (Zhu et al., 2010), we identified 234 NPR1-proximal proteins based on their enrichment in the NPR1–3 × HA-TurboID sample compared to the control (Fold Change_LFQ_ ≥ 2, *p* < 0.01 in either mild or harsh condition or *p* < 0.1 in both conditions) (Figure 1A and Supplemental Figure 1B; Supplemental Data 1). To validate our TurboID experimental results, we first examined the proximal partners for previously identified NPR1 interactors. We found that, while expressed at a similar level as the negative control protein, NPR1-TbID captured almost all known NPR1 interactors identified through decades of genetic and molecular studies, including NPR3 and NPR4 (Fu et al., 2012), NIM1-interacting 1 (NIMIN1) (Weigel et al., 2005), TGA5 (Zhang et al., 1999; Despres et al., 2000; Zhou et al., 2000), WRKY18 (Chen et al., 2019), histone acetyltransferase of the creb binding protein family 1 (Jin et al., 2018), and components of Mediator (Zhang et al., 2013) (Figure 1B), validating the specificity of the method. Critically, the identified proximal proteins show minimal overlap with the components of cSINCs (Zavaliev et al., 2020) (Figure 1C), giving us confidence that we identified the nuclear NPR1 proxiome, likely containing components of the NPR1 enhanceosome, instead of cSINCs, which form later with higher levels of SA.

This analysis also identified many new NPR1 proximal partners. Gene Ontology (GO) term analysis based on molecular function (MF) demonstrated that these partners are enriched with proteins involved in histone modifications, chromatin remodeling, transcriptional machinery, and splicing complexes (Figure 1D and Supplemental Figure 1C), suggesting involvement of these nuclear functions in reprogramming the SA transcriptome. The multi-functional feature of the NPR1 proxiome is consistent with its central role as a signaling hub for conferring disease resistance against a broad spectrum of pathogens and abiotic stresses (Olate et al., 2018; Seo et al., 2020; Zavaliev et al., 2020).

To validate newly identified NPR1-proximal complexes, we focused on two groups of NPR1 partners: (1) the chromatin remodeling switch/sucrose non-fermentable (SWI/SNF) proteins, with BRAHMA (BRM) as a representative, and (2) the histone modifying proteins, with the histone demethylase LSD1-like 3 (LDL3) as a representative. Although chromatin remodeling, nucleosome repositioning, and histone modifications have been shown previously to occur at SA-responsive genes and may play a role in their induction (Singh et al., 2015; Jin et al., 2018), the involvements of BRM and LDL3 have not been tested in SA-induced resistance. We first validated their associations with NPR1 using bimolecular fluorescence complementation (BiFC) in *Nicotiana benthamiana* and observed an SA-dependent increase in the associations between LDL3 or the C terminus of BRM and NPR1 (Figure 1E and Supplemental Figure 1D). With confirmation of their *in vivo* association, we then performed functional validation of their role in SA-mediated resistance. We found that knocking out the *BRM* and *LDL3* genes partially compromised SA-induced resistance to the bacterial pathogen *Pseudomonas syringae* pv. *maculicola* ES4326 (*Psm* ES4326), and complementation using the wild-type (WT) *BRM* (in *brm-1*) and *LDL3* genes restored the SA-induced resistance (Figure 1F and 1G and Supplemental Figure 1E and 1F), indicating that chromatin remodeling through BRM and histone demethylation by LDL3 are involved in SA-mediated defense. It is worth noting that, given the crucial roles of chromatin remodeling and histone modifications in general transcription regulation, the background effects of the *brm-3*, *ldl3–1*, and *ldl3–2* mutations had to be taken into consideration by comparing the mutant ± SA data with the WT ± SA data using a two-way ANOVA. The moderate but significant defense phenotypes of these mutants highlight the efficacy of TurboID in identifying core components of gene expression, which are normally difficult to uncover using forward genetic approaches due to their pleiotropic phenotypes or low viability.

Interestingly, both BRM and LDL3 proteins have been reported in proximity to the condensate-forming protein guanylate-binding protein-like 3 (GBPL3), which is involved in temperature-sensitive SA synthesis and pathogen response (Huang et al., 2021; Kim et al., 2022; Tang et al., 2022). From an in-depth comparison between the NPR1 proxiome and the GBPL3 proxiome, we discovered a large overlap in transcriptional regulators, chromatin remodelers, and histone modifiers (Figure 1H, shaded in blue). However, most of the TFs appeared to be NPR1-specific partners (19 of 24). This supports the hypothesis that transcriptome reprogramming is mediated by recruiting common transcriptional regulatory modules and machineries to unique TFs through hub proteins, such as NPR1, which have the intrinsic property to form biomolecular condensates (Mann and Notani, 2023).

### QuantSeq reveals that WRKY54 and WRKY70 are positive regulators of SA/NPR1-mediated transcriptional reprogramming

NPR1 is known to interact with several different TFs and TF families, including TGAs, WRKYs, teosinte branched 1/cycloidea/proliferating cell factors, myelocytomatosis, heat shock factors, and ethylene-insensitive 3 (Zhang et al., 1999; Saleh et al., 2015; Li et al., 2018; Olate et al., 2018; Huang et al., 2020; Nomoto et al., 2021). Among the TFs unique to NPR1 based on our TurboID data, TGA and WRKY TFs have been observed in multiple studies to interact with NPR1 in response to SA (Zhang et al., 1999; Despres et al., 2000; Zhou et al., 2000; Saleh et al., 2015; Chen et al., 2019; Zavaliev et al., 2020) (Figure 1B and 1H). While the TGA3 TF has been shown to bind DNA in complex with NPR1 in the cryoelectron microscopy structure (Kumar et al., 2022), the transcriptional role of WRKY TFs and their relationship with NPR1 in SA-mediated gene expression is less straightforward. WRKYs constitute a diverse TF family whose own expression is dynamically induced upon stress, displaying functional redundancies as well as distinct roles in gene expression regulation (Kalde et al., 2003; Wang et al., 2006; Xu et al., 2006). In this study, we focused on WRKY70 and its closest homolog, WRKY54, (WRKY54/70) because, although WRKY70 has been shown to associate with NPR1, its single mutant exhibits minimal transcriptional differences compared to WT plants (Saleh et al., 2015). We performed QuantSeq (Moll et al., 2014) on WT, *npr1–2*, and the *wrky54 wrky70* (*wrky54/70*) double mutant 8 h after SA induction. Principal-component analysis (PCA) demonstrated a separation of the WT treated with SA from all other samples (Supplemental Figure 2A), indicating that both *npr1–2* and *wrky54/70* exhibit abnormal responses to SA compared to the WT. In the WT, we identified 3528 differentially expressed genes in response to SA but only 722 and 532 in *npr1–2* and *wrky54/70,* respectively (|log_2_fold change| ≥ 1, adjusted *p* < 0.1) (Supplemental Figure 2B–2D; Supplemental Data 2). Furthermore, both mutants displayed few differentially expressed genes basally compared to the WT (Supplemental Figure 2E and 2F), indicating that the loss of induction by SA in the mutants is not due to variations in their background gene expression. Among the 1909 SA-induced genes, 1022 were NPR1 dependent, and 804 were WRKY54/70 dependent (Supplemental Figure 2G; Supplemental Data 2), and the global transcriptome displayed a higher degree of correlation with NPR1 than with WRKY54/70 (Supplemental Figure 2H and 2I). GO term analyses of NPR1-and/or WRKY-dependent genes did not provide further resolution, with similar enrichments for defense response and SA-related processes (Supplemental Figure 3A–3C). Interestingly, promoter examination of these genes led to the detection of the WRKY-binding “W box” as the most enriched motif (Supplemental Figure 3D–3F), instead of the *as-1* element for TGA TFs, even for those NPR1-dependent, WRKY54/70-independent genes (Supplemental Figure 3F), suggesting that WRKY TFs are the major TFs responsible for the SA-mediated transcriptional output.

### Genome-wide greenCUT&RUN identifies WRKY TF genes as a major group of NPR1 transcriptional targets

The enrichment of the W box in our QuantSeq data (Supplemental Figure 3D–3F) and in other transcriptome profiling datasets at various time points after SA or SA analog treatment (Maleck et al., 2000; Wang et al., 2006; Ding et al., 2018; Jin et al., 2018) (Supplemental Figure 3G–3I) raised the question of the role of TGA TFs in the SA signaling cascade and the relationship between TGA or WRKY TFs and NPR1. To address these questions, we performed cleavage under target and release using nuclease (CUT&RUN), followed by next-generation sequencing (Skene and Henikoff, 2017), on *35S:NPR1-GFP* and *35S:GFP* transgenic plants 4 h after SA induction to identify direct transcriptional targets of NPR1, utilizing an anti-GFP antibody. Since CUT&RUN does not require cross-linking, it offers a major advantage over the traditional chromatin immunoprecipitation sequencing (ChIP-seq) methods by reducing false positives introduced by cross-linking and allowing identification of loci bound by protein of interest in the native chromatin state (Meers et al., 2019b). Unfortunately, the experiment failed to detect any differential peaks between NPR1-GFP and GFP samples, with a minimal difference seen at either known NPR1 targets or globally (Supplemental Figure 4A–4D). This suggests that, while CUT&RUN has significantly enhanced sensitivity for identifying TFs that interact directly with chromatin (Meers et al., 2019a) and histone modifications (Zheng and Gehring, 2019), an even more sensitive methodology is required for detecting targets of transcriptional cofactors, like NPR1, whose proximity to DNA depends on its interaction with TFs.

**Figure 2.**
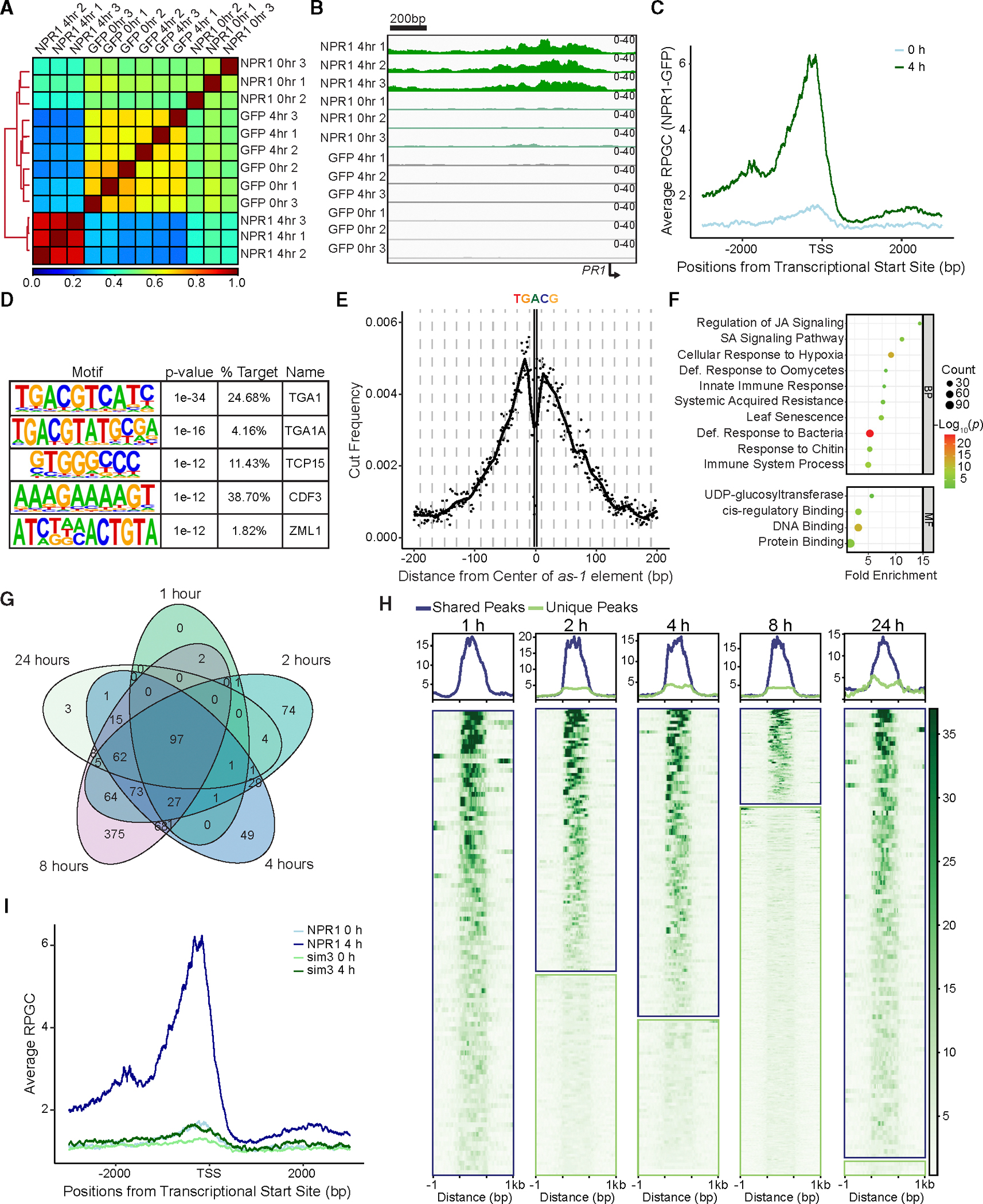
NPR1 targets TF gene promoters through association with TGA TFs. **(A)** Pearson’s correlation of the greenCUT&RUN data from plants expressing NPR1-GFP (NPR1) and GFP with and without 1 mM SA treatment for 4 h. **(B)** Integrative Genomics Viewer (IGV) view of the *PR1* promoter, showing normalized NPR1-GFP and GFP binding before and after SA treatment. **(C)** Mean profile of reads per genomic content (RPGCs) of NPR1-GFP reads before and after SA treatment at NPR1 target genes. TSS, transcription start site. **(D)** Motifs enriched under NPR1-GFP peaks 4 h after 1 mM SA treatment. **(E)** Cut frequency of all *as-1* elements (TGACG) by the GFP nanobody MNase in the overall NPR1-GFP peaks 4 h after 1 mM SA treatment. **(F)** The enriched biological processes (BPs) and MFs of NPR1 target genes. **(G)** Venn diagram displaying shared NPR1 loci 1, 2, 4, 8, and 24 h after 1 mM SA treatment. **(H)** Heatmaps and mean profile of normalized (RPGC) NPR1 binding at shared loci (outlined in blue) and unique loci (outlined in green) 1, 2, 4, 8, and 24 h after 1 mM SA treatment. **(I)** Mean profile of RPGC of NPR1-GFP and npr1^sim3^-GFP (sim3) before and after SA treatment at NPR1 target genes.

To further improve the sensitivity of the CUT&RUN methodology, which relies on transient interactions of multiple proteins that ultimately lead to the cutting and release of target DNA sequences by protein A-monococcal nuclease (pA-MNase), we adopted aGFP-tagged factor CUT&RUN approach, (greenCUT&RUN), where a GFP-specific nanobody is fused directly to the MNase (Koidl and Timmers, 2021). Similar to CUT&RUN, greenCUT&RUN also allows profiling of the chromatin in the native state to reduce the number of false positives (Nizamuddin et al., 2021). In contrast to the initial CUT&RUN data (Supplemental Figure 4A–4D), the new method led to a clear separation of the SA-treated NPR1-GFP samples from both the untreated NPR1-GFP and the GFP samples (Figure 2A). Further demonstrating the success of our greenCUT&RUN experiment, PCA showed a clear clustering of SA-treated NPR1 samples separated from all other samples (Supplemental Figure 4E). Based on the three NPR1-GFP replicates, we were able to detect 385 reproducible NPR1-GFP-specific peaks (Supplemental Figure 4F; Supplemental Data 3). Furthermore, by examining the promoter of the known NPR1 target gene, *PR1*, an SA-dependent accumulation of NPR1-GFP could clearly be observed compared to the GFP input (Figure 2B). By averaging the global alignment of the binding loci, we detected a significant enrichment of NPR1-GFP at the promoters of its target genes upon SA treatment compared to the untreated samples (Figure 2C). Among these loci, 84.2% occurred upstream of the transcriptional start site (TSS). Interestingly, the distances from the TSSs of these binding peaks varied widely from gene to gene, ranging from immediately before the TSS to several thousand base pairs upstream, with only 53% within 1 kb from the TSS (Supplemental Figure 4G). These results are consistent with the proposed function of NPR1 in organizing an enhanceosome by bridging distal binding sites through DNA looping and interacting with larger transcriptional machineries like the SWI/SNF complex and Mediator (Bazett-Jones et al., 1999; Kagey et al., 2010) (Figure 1H).

Among the NPR1 peaks, we detected the TGA-binding *as-1* element, TGACG, as the most significantly enriched motif (Figure 2D). While there was an increased cutting frequency by the MNase near the motif, the motif itself was protected, further supporting the notion that NPR1 binds to the DNA through TGA TFs (Figure 2E). Additionally, we also detected enrichment of teosinte branched 1/cycloidea/proliferating cell factors and cycling Dof factor binding motifs (Figure 2E), which are two other TFs detected in our TurboID experiment (Figure 1H). Further supporting the TGA binding motif being the most enriched NPR1 locus, analysis of previously published DNA affinity purification sequencing data (O’Malley et al., 2016) demonstrated that TGA5 binds to the same region as NPR1 (Supplemental Figure 5). As expected, the NPR1 target genes are largely related to defense response and cross-talk between SA and another plant defense hormone, jasmonic acid (Figure 2F).

The reduced sequencing depth needed for greenCUT&RUN allowed us to perform a time course on NPR1-GFP in response to SA. Analysis of the data detected many shared targets at all time points (Figure 2G). Interestingly, these shared peaks (97 loci) displayed stronger chromatin binding compared to the time point-specific peaks (Figure 2H). To compare methods, we then examined our greenCUT&RUN data 8 h after SA treatment with a recently reported NPR1 ChIP-seq performed after treatment with the synthetic analog of SA, 2,6-dichloroisonicotinic acid, for 10 h, followed by a mild or harsh chromatin isolation protocol (Yun et al., 2024). We observed 207 overlapping peaks between our greenCUT&RUN and both ChIP-seq conditions (Supplemental Figure 6A). The majority of unique peaks were observed in the mildly processed ChIP-seq (“mild-specific”) (Supplemental Figure 6A). While motif analysis of all samples showed enrichment of the *as-1* element (Supplemental Figure 6B–6D), the GO term analysis displayed striking differences, with the shared peaks enriched with defense-related biological processes (Supplemental Figure 6E), while the mild-specific peaks from ChIP-seq were largely enriched in response to other stresses (Supplemental Figure 6F). This is in contrast to the greenCUT&RUN-specific peaks, which still had GO terms related to SA signaling and defense (Supplemental Figure 6G). We hypothesize that these mild-specific signals in ChIP-seq may result from the cross-linking step, which could capture transient interactions between NPR1 and TGA, when NPR1 scans the chromosome, instead of the more stable and transcriptionally active TGA_2_-NPR1_2_-TGA_2_ enhanceosome complex (Kumar et al., 2022).

Interestingly, NPR1 does not display binding enrichment of the W box at any time after SA treatment (Supplemental Figure 7A). Furthermore, greenCUT&RUN on the NPR1 SUMO-deficient mutant, npr1^sim3^ (sim3), which preferentially interacts with WRKY70 (Saleh et al., 2015), showed minimal binding at NPR1 loci (Figure 2I), with no enriched motifs. Taken together, these results indicate that NPR1 is associated with TGA TFs, but not WRKYs, during the course of SA induction. Moreover, compared to the thousands of differentially expressed genes in response to SA, there were only a few hundred NPR1 target genes. These data suggest that NPR1 reprograms the transcriptome through multiple steps instead of through parallel association with multiple TFs. In support of this hypothesis, the GO terms of NPR1 transcriptional targets are largely enriched with TFs and other DNA-binding proteins (Figure 2F). Analysis of the genes annotated as DNA binding and/or *cis*-regulatory binding detected four major TF families: WRKYs, NAM ATAF and CUCs, ETHYLENE-RESPONSE FACTORs, and MYELOBALSTOSIS VIRAL ONCOGENE HOMO-LOGs, with WRKYs representing the largest family (Supplemental Figure 7B). Of note, NPR1 preferentially targets group III WRKY TFs, including WRKY70 (Supplemental Figure 7C and 7D), suggesting their involvement in further propagating SA-induced gene expression.

### Genome-wide greenCUT&RUN analysis identifies WRKY70 as a downstream TF in the SA-induced transcriptional cascade

**Figure 3.**
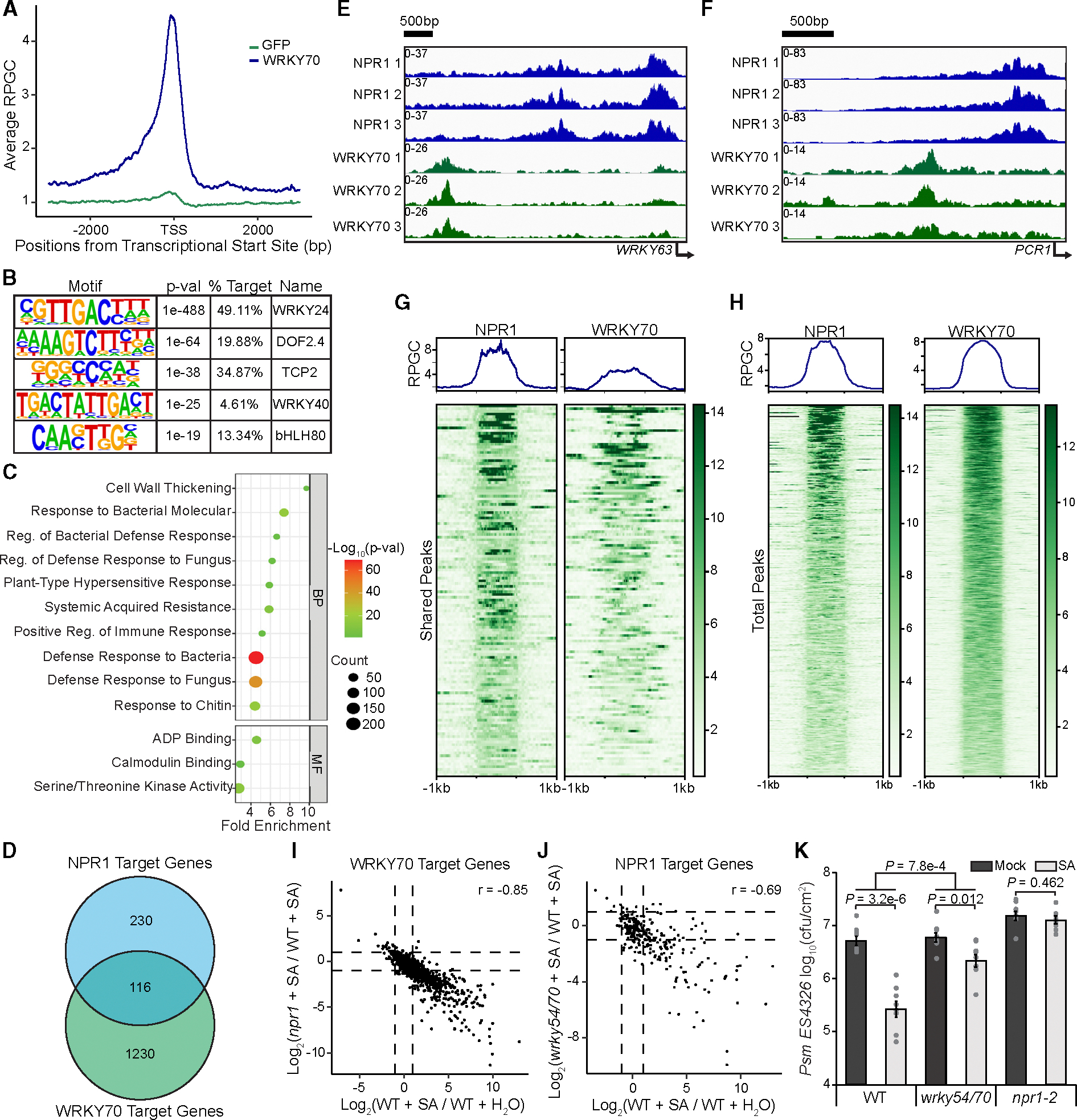
WRKY54/70 are major TFs downstream of NPR1-TGA that positively regulate SA-mediated gene expression and resistance. **(A)** Mean profile of RPGCs of WRKY70-GFP (WRKY70) and GFP reads of WRKY70 target genes. **(B)** Motifs enriched under WRKY70-GFP peaks. **(C)** Enriched BPs and MFs of WRKY70 target genes. **(D)** Venn diagram illustrating the overlap between NPR1 and WRKY70 target genes. **(E and F)** IGV view of normalized NPR1 and WRKY70 binding at the promoters of their shared target genes *WRKY63* **(E)** and *PCR1* **(F)**. **(G)** RPGCs of NPR1-GFP and WRKY70-GFP at 116 shared target genes 1 kb upstream and downstream of NPR1 peaks. **(H)** RPGCs of all NPR1-GFP and WRKY70-GFP target genes centered on their respective peaks. **(I)** Correlation between SA-induced transcription and NPR1 dependency in WRKY70 target genes. r, Pearson correlation coefficient. **(J)** Correlation between SA-induced transcription and WRKY54/70 dependency in NPR1-target genes. **(K)** Bacterial CFUs in WT, *wrky54/70*, and *npr1–2.* Plants were treated with H_2_O (mock) or 1 mM SA for 24 h before being inoculated with *Psm* ES4326 at OD_600_ = 0.001. CFUs were measured 2 days post inoculation (*n* = 8; error bars represent SEM; two-sided *t*-test and two-way ANOVA were used for comparison within and between genotypes, respectively).

To examine the role of group III WRKYs in SA/NPR1-mediated reprogramming of the immune transcriptome, we performed a subsequent greenCUT&RUN analysis on *35S:WRKY70*, the most abundantly expressed *WRKY* in the WT after SA treatment (Supplemental Data 2). Previously, WRKY70 has been hypothesized to be removed from the *PR1* promoter by NPR1 in response to SA (Saleh et al., 2015). To consider this hypothesis, we collected samples 2 h after SA treatment. Similar to our NPR1-GFP greenCUT&RUN experiment, we found that WRKY70-GFP samples were well correlated with one another but distinguished from GFP samples (Supplemental Figure 8A and 8B). Surprisingly, they were also distinct from the NPR1-GFP greenCUT&RUN data (Supplemental Figure 8A and 8B). From this experiment, we detected 1477 reproducible WRKY70-GFP-specific peaks (Supplemental Figure 8C; Supplemental Data 4). It was evident that the WRKY70-GFP samples had a higher percentage of reproducible peaks (43.4%–61.3%) compared to those in the NPR1-GFP samples (33.1%–36.3%) (Supplemental Figures 4F and 8C), consistent with WRKY70 being a TF binding directly to DNA. Examining all target genes showed that WRKY70, like NPR1, was mainly detected at the promoters of its target genes, with only 14.4% of WRKY70 >1 kb upstream of the TSS compared to the 31.2% for NPR1 (Figure 3A and Supplemental Figures 4G and 8D). As expected, a high enrichment of the W box was observed in these WRKY70-bound loci (Figure 3B). Interestingly, while defense-related biological processes were still the top enrichments in the WRKY target genes, they differ from those of NPR1-target genes in their MFs. Where NPR1 targets TF genes, WRKY70 targets those involved in ADP binding (mostly encoding nucleotide-binding domain and leucine-rich repeat-containing immune receptors), calmodulin-binding, and kinase activity (Figure 3C), implying that WRKY70, whose transcription is induced by NPR1-TGA (Wang et al., 2006) (Supplemental Data 2), is involved in the downstream events in the signaling cascade of NPR1-mediated transcriptional reprogramming. Furthermore, these genes were highly correlated to those transcriptionally impaired in the *wrky54/70* mutant (Supplemental Figure 8E), with ~49% (522 of 1067) of WRKY70 targets with detectable expression being differentially expressed in response to SA and ~68% (355/522) displaying differential expression dependency on WRKY54/70 (Supplemental Data 2 and 4), demonstrating that they are true WRKY70 targets.

Apart from these distinct transcriptional targets, there were a smaller number of shared target genes between WRKY70 (116 of 1476) and NPR1 (116 of 346) (Figure 3D), suggesting a possible interplay between WRKY70 and NPR1 in regulating the transcription of these genes. However, in two common target promoters examined, we detected WRKY70 and NPR1 at distinct loci from one another (Figure 3E and 3F). Interestingly, *PR1* was not even detected in our WRKY70-GFP samples, despite the negative regulation WRKY70 exerts on the transcript (Li et al., 2017). To further examine the relationship between NPR1 and WRKY70, we examined the peak patterns at all shared target gene promoters. As expected, NPR1 samples showed one distinct peak (Figure 3G), typical of its global target profile (Figure 3H), while WRKY70 samples were not confined to the defined peak region of NPR1, displaying binding near but not in the peak region (Figure 3G), which is atypical for its own global target profile, where WRKY70 binding is confined within the peak region (Figure 3H). These data further demonstrate that NPR1 is unlikely to switch associations between WRKY and TGA TFs at the chromatin level, as proposed previously (Saleh et al., 2015). Instead, NPR1 has been found to interact with WRKY70 in cSINCs to sequester and degrade WRKY70 (Zavaliev et al., 2020). Nevertheless, the shared gene targets of NPR1 and WRKY70 with distinct loci suggest a possible regulatory dependence on both proteins.

The sequential NPR1- and WRKY70 greenCUT&RUN analyses elucidated an SA signaling cascade in which the SA-activated NPR1 induces the expression of *WRKY* TF genes through association with TGA TFs. Consistently, by comparing our QuantSeq results with NPR1 and WRKY70 greenCUT&RUN targets, we found that, while NPR1 had the expected strong regulation of WRKY70 target genes (r = 0.85) (Figure 3I), WRKY54 and WRKY70 had a more moderate correlation with NPR1 targets (r = 0.69) (Figure 3J), demonstrating that WRKY54/70 targets are regulated by NPR1, while NPR1 targets are also regulated by WRKY54/70, likely through a feedback loop. Taken together, these results suggest that WRKY54 and WRKY70 are predominantly positive TFs of SA-mediated gene transcription, in addition to their role as feedback repressors of SA synthesis (Wang et al., 2006). This hypothesis is further supported by the compromised SA-mediated resistance to *Psm* ES4326 observed in the *wrky54/70* double mutant compared to the WT (Figure 3K).

### SA-induced condensate formation of NPR1 promotes its chromatin binding and transcriptional activity

**Figure 4.**
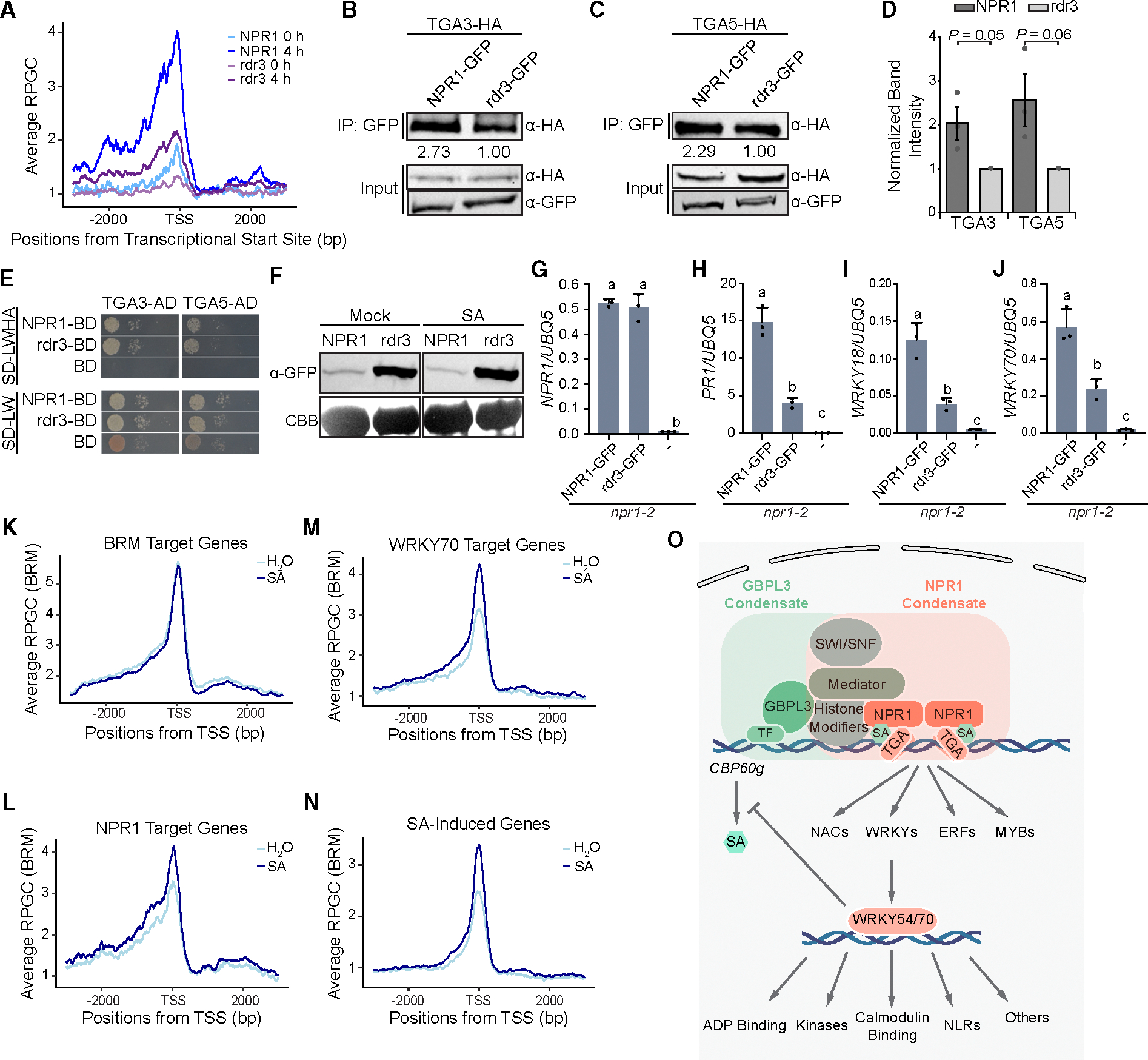
Biomolecular condensate formation stabilizes NPR1 association with the TGA TF and enhances its transcriptional activity. **(A)** Mean profile of RPGC of NPR1-GFP (NPR1) and npr1^rdr3^-GFP (rdr3) at NPR1 target genes before and after 4 h of 1 mM SA treatment. **(B and C)** CoIP between TGA3 **(B)** or TGA5 **(C)** and NPR1 or rdr3 transiently overexpressed in *N. benthamiana*. The value under the IP blot represents band intensities normalized to TGA TF input. **(D)** Quantification of normalized coIP TGA3-HA or TGA5-HA band intensity from three independent replicates (*n* = 3, error bars represent SEM; two-sided *t*-test was used for comparison between NPR1 and rdr3). **(E)** Interaction between TGA3 or TGA5 fused to the activator domain (AD) and NPR1 or rdr3 fused to the DNA-binding domain (BD) in the yeast two-hybrid assay. Yeast strains were mated for 24 h, normalized to OD_600_ = 1.0, serially diluted, and plated on the indicated synthetic defined (SD) medium without leucine and tryptophan (LW) or without leucine, tryptophan, histidine, and adenine (LWHA) and incubated at 30°C. Photos were taken 2 days after plating. **(F)** Protein level of NPR1-GFP and rdr3-GFP in stable transgenic *Arabidopsis* plants. **(G–J)** Transcript levels of *NPR1* or *rdr3* **(G)** and the target genes *PR1* **(H)**, *WRKY18* **(I)**, and *WRKY70* **(J)** in *35S:NPR1-GFP/npr1–2*, *35S:npr1*^*rdr3*^*-GFP/npr1–2*, and *npr1–2* plants measured using qPCR 8 h after SA induction (*n* = 3, error bars represent standard deviation). **(K–N)** Mean profile of RPGCs of BRM-GFP reads at BRM target genes **(K)**, NPR1 target genes **(L)**, WRKY70 target genes **(M)**, and SA-induced genes **(N)** 4 h after treatment with H_2_O or 1 mM SA. **(O)** Working model of the SA/NPR1 signaling hub and transcriptional cascade. Overlapped rectangular shades show that NPR1 and GBPL3 condensates share general transcriptional regulatory machineries (e.g., Mediator, SWI/SNF, and histone modifiers) but target different genes through association with unique TFs. An increase in SA level triggers the transcriptional cascade by first activating NPR1 to induce TGA-mediated expression of WRKY, MYB, NAC, and ERFTFs, which, in turn, activate the subsequent gene expression.

With the identification of NPR1 proximal partners and direct transcriptional targets in the signaling cascade, we then tested our hypothesis that SA-induced condensate formation is critical for NPR1 to organize the enhanceosome to initiate transcription. We first performed greenCUT&RUN in a transgenic line expressing the npr1^rdr3^-GFP protein (referred to as rdr3) (Zavaliev et al., 2020), which accumulates to high levels in the nucleus upon SA induction but fails to form either nuclear or cytoplasmic condensates (Zavaliev et al., 2020). We found that chromatin association of rdr3 occurred at the same loci as the WT NPR1 in an SA-dependent manner but at a significantly lower affinity (Figure 4A), even though the mutant protein has a higher-than-WT nuclear distribution (Zavaliev et al., 2020). Interestingly, the reduced rdr3 binding to the TGA TFs was only observed *in planta* (Figure 4B–4D), not in the yeast two-hybrid assay (Figure 4E), suggesting that the decreased rdr3 chromatin association is less likely to be due to its diminished binding to TGA TFs than the reduced stability of its complex with TGA TFs due to inability to form the nuclear condensates. Moreover, despite elevated protein amounts and comparable transcript levels (Figure 4F and 4G), rdr3 exhibited significantly compromised activity in inducing direct target genes, *PR1*, *WRKY18*, and *WRKY70* (Figure 4H–4J), compared to the WT NPR1-GFP control, supporting our hypothesis that NPR1 orchestrates the transcriptomic changes upon SA induction by forming biomolecular condensates.

### BRM association with NPR1 and WRKY70 targets is enhanced upon SA treatment

Our TurboID experiment identified BRM in the NPR1 nuclear proxiome (Figure 1H), and loss of BRM resulted in compromised SA-induced resistance (Figure 1F and Supplemental Figure 1E). However, due to the essential role that BRM plays in transcription, it is unclear whether its proximity to NPR1 is a specific mechanism for activating NPR1 target genes. To address this question, we performed greenCUT&RUN on *BRM:BRM-GFP* transgenic plants (Li et al., 2016) treated with 1 mM SA or H_2_O for 4 h. Interestingly, we found that the overall BRM accumulation stayed constant under both mock and SA-induced conditions at BRM-specific peaks, indicating that SA has minimal impact on general BRM binding to chromatin (Figure 4K; Supplemental Data 5). However, in agreement with our TurboID and BiFC data (Figure 1E and 1H), we observed an increase in BRM accumulation at NPR1 loci upon SA treatment (Figure 4L). Interestingly, we also detected a similar increase at WRKY70 loci (Figure 4M), indicating that BRM is present at these loci, not as a signaling mechanism, but as a component of the common transcriptional machinery. Consistent with this hypothesis, increased levels of BRM were also observed at SA-induced genes upon SA treatment (Figure 4N). It would be interesting to examine other regulatory hubs to see whether they also have similar proteins in their proximity, thus allowing comprehensive mapping of general transcriptional machinery.

## DISCUSSION

By combinatorial applications of label-free quantification of TurboID-based LC-MS/MS data and the greenCUT&RUN technology, for the first time in plants, we transcended decades of molecular genetic studies to generate a comprehensive map of the NPR1-centered transcriptional reprogramming machineries and the transcriptional cascade in response to SA induction (Figure 4O). The validation of the new NPR1 proximal partners (Figure 1E–1G and Supplemental Figure 1E and 1F) clearly demonstrates the effectiveness of the methodology in identifying signaling hubs formed by proteins, like NPR1, in association with regulatory modules involved in common nuclear functions, such as chromatin remodeling, histone modifications, Mediator, and RNA splicing, that also play roles in other stress responses. The robustness of these essential cellular machineries makes it difficult to discern their contributions to specific biological processes through genetic studies. Indeed, the NPR1 proxiome shows high similarity to the GBPL3 proxiome (Tang et al., 2022), with the major distinction being in their associated TFs (Figure 1H). Since both the GBPL3 proxiome, involved in inducing SA synthesis upon stress (Kim et al., 2022), and the NPR1 proxiome, responsible for SA-mediated transcriptional reprogramming, can form detectable nuclear biomolecular condensates when overexpressed (Saleh et al., 2015; Huang et al., 2021), it is tempting to hypothesize that, in the nucleus, a similar set of transcriptional regulatory modules is recruited to form supramolecular complexes/condensates by distinct regulators, like NPR1, whose association with unique TFs provides the complex/condensate functional specificity (Figures 1H and 4O). Furthermore, condensate formation facilitates NPR1’s association with chromatin as well as target gene induction (Figure 4A–4J), supporting the notion that SA-induced nuclear NPR1 condensates (i.e., SA-induced NPR1 nuclear condensates), are transcriptionally active.

More experiments are required to demonstrate that NPR1 condensate formation is required for the recruitment of the transcriptional regulatory modules identified in the NPR1 proxiome (Figure 1A and Supplemental Figure 1B; Supplemental Data 1). Though the BRM greenCUT&RUN showed an increase in association to NPR1 loci upon SA induction, significant basal signal was detected in the absence of SA (Figures 1E and 4L), indicating that the association of BRM to NPR1 loci is unlikely to be SA dependent but, rather, SA stabilized. It would be exciting to explore which proteins of these transcriptional modules are constitutively present at the promoters and which are recruited in response to induction to initiate transcription. Consistent with NPR1 condensate formation being a dynamic process, SA/NPR1-induced WRKYs as well as several known negative regulators of SA-mediated gene expression, such as NPR3, NPR4, NIMINs, and TPLs, were found to be in the NPR1 proxiome. However, we cannot rule out the possibility that the NPR1 proxiome consists of multiple distinct NPR1-protein complexes or represents responses in different subcellular compartments or leaf cell types (Delannoy et al., 2023; Nobori et al., 2023; Zhu et al., 2023). Future research will be required to understand the dynamics of the NPR1 signaling hub.

The advantage of the greenCUT&RUN method in detecting dynamic chromatin association in the native state is critical for avoiding false positives in identifying true targets of a regulator, like NPR1, which must scan the chromatin to find its partners for productive binding (Kumar et al., 2022). The ~10 fold-increase in sensitivity compared to ChIP-seq and the ease of the greenCUT&RUN method make a time-course experiment feasible, which, in this study, identified not only the direct NPR1 targets but also the hierarchical relationship between TGA and WRKY TFs, demonstrating its great potential in dissecting transcriptional cascades. The method has also been shown to be effective in studying dynamic TFs, like WRKY54/70, which play a positive role in SA signaling (Figure 3), in addition to their negative role in SA synthesis reported in our previous study (Wang et al., 2006). The requirement of WRKY54/70 for SA-mediated gene expression and defense discovered in this genome-wide study serendipitously solved the puzzle of why the high levels of SA in the *wrky54/70* double mutant do not lead to *PR* gene induction or enhanced disease resistance (Wang et al., 2006) (Figure 4O).

The SA-responsive TF hierarchy unveiled through the stepwise greenCUT&RUN was previously obscured in the transcriptomic data. As demonstrated in our QuantSeq experiments, statistical analyses of such data failed to detect the initiation step of the SA signaling cascade mediated by NPR1/TGA due to the overwhelming number of WRKY-mediated transcriptional targets induced in the subsequent step of the cascade. Moreover, transcriptomic studies of TF gene families often rely on the usage of available TF knockdown lines or knockout mutants, which either have weak phenotypes due to functional redundancy or pleiotropic defects when higher-order mutants are used. These limitations can now be overcome by the greenCUT&RUN method, which is readily applicable for studying not only TFs but also proteins with indirect chromatin association.

## METHODS

### Plant material and growth conditions

All plants used in this study were grown on soil (ProMix B) under 12-h light/12-h dark conditions. The *35S:YFP-YFP-TbID* line was generously gifted by Dr. Zhi-Yong Wang (Carnegie Institution for Sciences) (Kim et al., 2023). The *35S:NPR1–3xHA-TbID* construct was made using Gateway cloning (Thermo Fisher Scientific). *35S:NPR1–3xHA-TbID* was transformed into *npr1–2* plants using the floral dip method (Clough and Bent, 1998). The *brm-3* (SALK_088462) and *ldl3–2* (SALK_146733) mutants were obtained from ABRC. The *35S:NPR1-GFP*, *35S:npr1*^*rdr3*^*-GFP*, and *35S:WRKY70-GFP* transgenic lines and the *wrky54 wrky70* double mutant have been described previously (Wang et al., 2006; Zavaliev et al., 2020). The *BRM:BRM-GFP* line was a generous gift from Dr. Chenlong Li (Sun Yat-sen University) (Li et al., 2016). The *LDL3:LDL3–3xFLAG* and *ldl3–1* lines were generous gifts from Dr. Tetsuji Katutani (University of Tokyo) (Mori et al., 2023).

### RNA isolation and qPCR

Total RNA was extracted from 3-week-old plants treated with 1 mM SA or H_2_O using Trizol (Rio et al., 2010) (Thermo Fisher Scientific). DNase-treated total RNA was then used for SuperScriptIII reverse transcription (Thermo Fisher Scientific). The resulting cDNA samples were diluted 10-fold for qPCR reactions using SYBR Green Master Mix to detect transcript levels.

### Affinity purification of biotinylated proteins

Affinity purification of biotinylated proteins was performed as described previously (Mair et al., 2019), with minor modifications. Briefly, three replicates (4 g/sample) of 3-week-old plants treated first with 1 mM SA and, 1 h later, with 50 mM biotin for 3 h were collected, flash frozen, and stored at −80°C. Samples were ground to a fine powder, dissolved in 4 ml of the extraction buffer (50 mM Tris-HCl [pH 7.5], 150 mM NaCl, 0.1% SDS, 1% NP-40, 0.5% Na deoxycholate, 1 mM EGTA, 1 mM DTT, and protease inhibitor cocktail), filtered, and sonicated. Sonicated samples were centrifuged, and biotin was removed from the resulting protein solution using PD-10 desalting columns (GE Healthcare). The flowthrough was collected and subjected to affinity purification using streptavidin beads (Thermo Fisher Scientific). The resulting samples on the streptavidin beads were processed under two conditions: harsh and mild. The harsh condition involved washing the beads twice with extraction buffer, once with 1 M KCl, once with 100 mM Na_2_CO_3_, once with 2 M urea in 10 mM Tris-HCl (pH 8), and twice again with extraction buffer. The mild condition involved washing the beads seven times with extraction buffer. The processed beads from both conditions were resuspended in 1 ml of extraction buffer for further processing. Prior to trypsin digestion, the beads underwent further washes. The bead samples corresponding to the harsh conditions were followed by harsh washes consisting of washing once with cold 1 M KCl, once with 2 M urea in 10 mM Tris-HCl (pH 8), twice with cold 50 mM Tris-HCl (pH 7.5), and twice with urea wash buffer (50 mM Tris-HCl [pH 7.5] and 1 M urea). The bead samples corresponding to the mild conditions were followed by mild washes consisting of washing seven times with PBS buffer. Both sample sets were subjected to a 3-h incubation in 80 μl trypsin buffer (50 mM Tris-HCl [pH 7.5], 1 M urea, 1 mM DTT, and 0.4 μg trypsin) at 25°C. The supernatants from the tryptic digest were transferred to new tubes, and the beads were washed twice with 60 μl1M urea in 50 mM Tris-HCl (pH 7.5). The combined 200-μl elutes were reduced (final concentration of 4 mM DTT), alkylated (final concentration of 10 mM iodoacetamide), and digested overnight with 0.5 μg trypsin. Additional 0.5 μg of trypsin was added the next morning, followed by acidification 4 h later by adding formic acid to a final concentration of ~1% and desalting using OMIX C18 pipette tips (A57003100).

### LC-MS/MS

LC-MS/MS was carried out on a Q-Exactive HF Hybrid Quadrupole-Orbitrap mass spectrometer (Thermo Fisher Scientific) equipped with an Easy LC 1200 UPLC LC system (Thermo Fisher Scientific). Peptides were first trapped using a trapping column (Acclaim PepMap 100 C18 High-Performance LC, 75-μm particle size, 2-cm bed length) and then separated using an analytical column (AUR2–25075C18A, 25CM Aurora Series Emitter Column, 25 cm × 75 μm, 1.6 μm C18, IonOpticks). The flow rate was 300 nl/min, and a 120-min gradient was used. Peptides were eluted by a gradient from 3% to 28% solvent B (80% acetonitrile, 0.1% formic acid) over 100 min and from 28% to 44% solvent B over 20 min, followed by a 10 min wash in 90% solvent B. Precursor scan was from *m/z* 375 to 1600, and the top 20 most intense multiply charged precursors were selected for fragmentation. Peptides were fragmented with higher-energy collision dissociation with normalized collision energy 27.

### Proteomic analysis

Harsh and mild sets of LC-MS/MS spectra were searched separately against the Araport11 database (September 14, 2022 version containing 49 467 entries) using the MSFragger 3.2 (Kong et al., 2017) software under default criteria to obtain maximum label-free quantification (MaxLFQ) intensities. The search results were analyzed separately in Perseus (v.1.6.15.0) (Tyanova et al., 2016). The processing in Perseus was as follows: MaxLFQ intensities were log2 transformed. Only proteins that had at least two valid values in at least one group (NPR1-TbID or YFP-YFP-TbID) were kept. The remaining missing MaxLFQ intensities were then imputed from a normal distribution that was downshifted by 1.8 and a width of 0.3 column wise. A two-sample *t*-test was conducted with a permutation-based (*n* = 250) false discovery rate (FDR) = 0.01 and S0 = 2. Significant NPR1 proximal partners were identified by the following criteria: (1) *p* < 0.1 in both processing conditions and NPR1_LFQ_/YFP_LFQ_ ≥ 2 or (2) *p* < 0.01 in either processing condition and NPR1_LFQ_/YFP_LFQ_ ≥ 2. GO term analysis was performed using Protein Analysis Through Evolutionary Relationships (PANTHER) (Mi et al., 2013). The interaction network was performed using Search Tool for the Retrieval of Interacting Genes/Proteins (Szklarczyk et al., 2019). Plots were generated with ggplot2 (Wickham, 2016), Cytoscape (Shannon et al., 2003), and SRplot.

### BiFC and analysis

*Agrobacterium* GV3101 carrying the indicated constructs was resuspended to optical density 600 (OD_600_) = 0.2 and OD_600_ = 0.8 for HTB1-mCherry and BiFC constructs, respectively, in acetosyringone-containing water (200 μM) before infiltrating the fully expanded *N. benthamiana* leaves. After 36 h, the leaves were treated with water (mock) or 1 mM SA for 8 h, followed by confocal imaging on a Carl Zeiss 880 Airyscan inverted confocal laser scanning microscope. A 488-nm argon laser was used to excite the YFP signal with a 516- to 544-nm emission filter, and a 561-nm diode-pumped solid-state laser was used to excite the mCherry signal with a 592- to 629-nm emission filter. Region of interest manager and BiFC intensities were measured using ImageJ (Schneider et al., 2012).

### SA-induced resistance against bacterial infection

SA-induced resistance was measured as described previously (Liu et al., 2015). Briefly, *Psm* ES4326 was grown at 30°C on plates containing King’s B medium for 48 h before being resuspended in 10 mM MgCl_2_. Three-week-old plants were pretreated with 1 mM SA or H_2_O for 24 h prior to infection with *Psm* ES4326 at OD_600_ = 0.001. Leaf discs from 8 infected plants were collected 2 days (for *wrky54 wrky70*) or 3 days (for *brm-3*, *BRM:BRM-GFP*, *ldl3–1*, *ldl3–2*, and *LDL3:LDL3–3* × *FLAG*) post infection and ground individually in 0.5 ml of 10 mM MgCl_2_, serially diluted, and plated on King’s B medium supplemented with 100 μg/ml streptomycin. Colonies were counted 2 days later.

### QuantSeq and data analysis

Total RNA was extracted from 3-week-old leaves treated with 1 mM SA or H_2_O for 8 h using the Split RNA Extraction Kit (Lexogen). RNA concentration was measured with the Qubit RNA BR assay (Thermo Fisher Scientific), and integrity was checked with an Agilent Technologies 2100 Bioanalyzer. Approximately 400 ng of RNA was used for library construction using the QuantSeq 3’ mRNA Seq Library Prep FWD Kit for Illumina (Lexogen) (Moll et al., 2014). All libraries were sequenced at 100-bp single-end reads using the Illumina NextSeq1000 system. Raw reads were trimmed to 50 bp using Trim Galore (Martin, 2011) and mapped to the TAIR10 genome using Spliced Transcripts Alignment to a Reference (Dobin et al., 2013) under the Lexogen-recommended parameters. Differential expression between SA- and H_2_O-treated samples was detected using DESeq2 (Love et al., 2014) with adjusted *p* < 0.1 and fold change ≥ 2. GO term analysis was performed using PANTHER (Mi et al., 2013), and *de novo* motif enrichment was uncovered using Hypergeometric Optimization of Motif EnRichment (Heinz et al., 2010) by analyzing promoters of differentially expressed genes from 1000 bp upstream to 200 bp downstream of the TSSs.

### greenCUT&RUN

Six leaves from two plants were collected before and after treatment with 1 mM SA for 4 h and stored at −80°C. Frozen samples were ground to a fine powder and dissolved in 15 ml of lysis buffer (20 mM Tris-HCl [pH 7.5], 20% glycerol, 20 mM KCl, 2 mM EDTA, 2.5 mM MgCl_2_, 8.56% sucrose, and protease inhibitor cocktail). Samples were filtered sequentially through a 70-μm filter and a 40-μm filter before centrifugation at 1500 *g* at 4°C for 10 min. The pellet was resuspended in the nucleus isolation buffer (20 mM Tris-HCl [pH 7.5], 20% glycerol, 2.5 mM MgCl_2_, 0.2% Triton X-100, and protease inhibitor cocktail) and centrifuged at 1500 *g* at 4°C for 10 min. These resuspension and centrifugation steps were repeated four times until the pellet was free of any green color. The pellet was resuspended in 1 ml of greenCUT&RUN wash buffer (20 mM 4–2-Hydroxyethyl) piperazine-1-ethane-sulfonic acid [HEPES]-KOH [pH 7.5], 150 mM NaCl, 0.5 mM spermidine, and protease inhibitor cocktail). Isolated nuclei were then bound to 40 μl of concanavalin A beads resuspended in 10 μl of binding buffer (20 mM HEPES-KOH [pH 7.5], 10 mM KCl, 1 mM CaCl_2_,1 mM MnCl_2_, and protease inhibitor cocktail) and rotated for 10 min at room temperature. The beads were collected using a magnetic rack. The supernatant was then removed, and the bound nuclei were resuspended in 1 ml of EDTA buffer (20 mM HEPES-KOH [pH 7.5], 150 mM NaCl, 0.5 mM spermidine, 2 mM EDTA, and protease inhibitor cocktail) and rotated at room temperature for 10 min. The beads were collected again, resuspended in 100 μl of greenCUT&RUN wash buffer containing 10 μg/ml of nanobody MNase (purified from *E. coli* strains carrying Addgene plasmid 166035 through fast protein LC), and rotated at 4°C for 30 min. After rotation, beads were collected and washed twice in greenCUT&RUN wash buffer. Beads were then put on ice, resuspended in 150 μL of calcium buffer (20 mM HEPES-KOH [pH 7.5], 150 mM NaCl, 0.5 mM spermidine, 3 mM CaCl_2_, and protease inhibitor cocktail), and incubated on ice for 30 min. After incubation, 100 μl of 2× stop buffer (340 mM NaCl, 20 mM EDTA, 10 mM EGTA, 100 μg/ml RNase A, and 50 μg/ml glycogen) was added to the beads and incubated at 37°C for 30 min. After incubation, beads were removed, and the supernatant was collected for DNA isolation. 2 μl of 10% SDS and 20 μg of Proteinase K were added to the collected supernatant and incubated at 50°C for 1 h. An equal volume of phenol:chloroform:isoamyl alcohol (25:24:1 [v/v]) was added to the samples, followed by vortexing. The solution was transferred to a phase lock tube and centrifuged for 5 min at 16 000 *g* at room temperature. After centrifugation, an equal volume of chloroform was added, and samples were inverted 10 × and centrifuged for 5 min at 16 000 *g* at room temperature. The top aqueous layer was then taken and moved into new tubes containing 3 μl of 2 mg/ml of glycogen. Two volumes of 100% ethanol were added to each sample to facilitate DNA precipitation overnight at −20°C. After DNA precipitation, samples were centrifuged for 10 min at 16 000 *g* at 4°C. The supernatant was removed, and the pellet was washed in 1 ml of 100% ethanol and centrifuged for 5 min at 16 000 *g* at 4°C. The supernatant was removed, and the pellet was air dried for 5–10 min. The pellet was resuspended in 50 μL of H_2_O and used for library preparation. The full protocol is available in the supplemental information.

### CUT&RUN

The nucleus isolation for the CUT&RUN protocol was the same as for greenCUT&RUN described above. After nucleus isolation, the previously reported CUT&RUN protocol (Skene and Henikoff, 2017) was followed.

### Construction and sequencing of the greenCUT&RUN and CUT&RUN libraries

The greenCUT&RUN and CUT&RUN libraries were constructed using the KAPA HyperPrep Kit (Roche), with minor modifications. Briefly, end repair and A tailing were performed at 20°C for 30 min, followed by deactivation of the A-tailing enzyme at 58°C for 1 h. Illumina TruSeq DNA UD Indexes diluted 1/100 were ligated onto A-tailed DNA at 20°C for 30 min. Post-ligation cleanup was performed twice, first using a 1 × library volume of AM-Pure Beads, nextwith a 1.2 × library volume of AMPure Beads, followedby a double-sided size selection to remove larger DNA fragments and smaller adapter dimers, respectively, using a 0.7–1.2× library volume of AMPure Beads following the manufacturer’s protocol (Roche). Ligated libraries were then amplified using PCR and cleaned up twice with a 1.2 × library volume of AMPure Beads to generate final purified libraries. Library size and concentration were determined using the Agilent Technologies 2100 Bioanalyzer and Qubit (Thermo Fisher Scientific), respectively. The *35S:NPR1-GFP*, *35S:npr1*^*rdr3*^*-GFP*, *35S:WRKY70-GFP*, and *35S:GFP* (control) libraries were sequenced at 75-bp paired-end reads using the Illumina NextSeq500 system. The *BRM:BRM-GFP* and *35S:GFP* (control) libraries were sequenced at 100-bp paired-end reads using the Illumina Next-Seq1000 system.

### CUT&RUN and greenCUT&RUN data analysis

Raw reads were trimmed using Trim Galore (Martin, 2011) and aligned to the TAIR10 genome using bowtie2 (Langmead and Salzberg, 2012). Concordant read sequence alignment map files were converted to binary alignment map files, and PCR-duplicated reads were removed using SAMtools (Li et al., 2009). Deduplicated binary alignment map (BAM) files were then used to call peaks using MACS2 (Zhang et al., 2008). Peaks called in all samples were used for further analysis. Bigwig and bedgraph files of normalized reads per genomic content (RPGC) were generated using bamCoverage from deepTools 3.5.1 (Ramirez et al., 2014). Bigwig files were visualized in Integrative Genomics Viewer (IGV) (Robinson et al., 2011). Normalized bigwig files and deepTools 3.5.1 were used for generating Pearson correlation heatmaps, PCA plots, and peak heatmaps. *De novo* motif prediction of reproducible peaks was performed using HOMER (Heinz et al., 2010). GO term analysis was performed using PANTHER (Mi et al., 2013). The cut frequency plot was generated using cut frequency (Nizamuddin et al., 2021). Mean profile plots were generated using custom code in R.

### Yeast two-hybrid assay

The AH109 and Y187 yeast strains were transformed with the TGA/pGADT7 and NPR1/pGBKT7 constructs, respectively. NPR1 and npr1^rdr3^ were used as bait, and TGA3 and TGA5 were used as prey. All protocols were carried out according to the Clontech Yeast Protocols Handbook.

### Protein analysis and immunoprecipitation

Protein analysis and immunoprecipitation IP were performed as described previously (Du et al., 2013). Briefly, recombinant proteins were transiently overexpressed in *N. benthamiana* by coinjecting *Agrobacterium tumefaciens* strain GV3101 carrying the *35S:NPR1-GFP* construct (OD_600_ = 0.5) or *35S:npr1*^*rdr3*^*-GFP* construct (OD_600_ = 0.5) with *A. tumefaciens* strain GV3101 carrying the *35S:TGA3-HA* or *35S:TGA5-HA* construct (OD_600_ = 0.5) into the abaxial side of the leaf. After 44 h, plants were sprayed with 1 mM SA for 4 h before 1 g of tissue was collected and flash frozen. Frozen tissue was then ground and resuspended in 2.5 ml of IP buffer (10% glycerol, 25 mM Tris-HCl [pH 7.5], 150 mM NaCl, 10 mM DTT, protease inhibitor cocktail, and 0.2% NP-40). Forty microliters of α-GFP beads (Chromotek) were added to the lysate for protein binding overnight at 4°C, followed by three washes in IP buffer. Fifty microliters of 4 × lithium dodecyl sulfate sample buffer (Thermo Fisher Scientific) was added to the beads and incubated at 70°C for 20 min. Samples were then run on a 4%–12% BisTris gel and transferred to a membrane for western blotting using α-GFP (Clontech) and α-HA (Cell Signaling Technology) antibodies. Band intensity was measured using the iBright analysis software (Thermo Fisher Scientific).

### Statistics and reproducibility

For all statistical data, the center values are the mean, and the error bars represent the standard error of the mean except for the qPCR data in Figure 4 (standard deviation). All experiments were performed three or more times with similar results, except the affinity purification LC-MS/MS, QuantSeq, and greenCUT&RUN, where one experiment with multiple biological replicates was performed.

## Supplementary Material

Powers et al., Supplemental information

Supplemental Data 1

Supplemental Data 2

Supplemental Data 3

Supplemental Data 4Supplemental Data 4

Supplemental Data 5

## Data Availability

The greenCUT&RUN, CUT&RUN, and QuantSeq sequencing data are available through the NCBI under accession number PRJNA1 050222. The MS proteomics data have been deposited into the ProteomeXchange Consortium via the PRIDE (Perez-Riverol et al., 2022) partner repository with the dataset identifier PXD047543. Code generated in this study is available on GitHub (https://github.com/jjp55).

## References

[R1] Bazett-Jones DP, Côté J, Landel CC, Peterson CL, and Workman JL (1999). The SWI/SNF complex creates loop domains in DNA and polynucleosome arrays and can disrupt DNA-histone contacts within these domains. Mol. Cell Biol. 19:1470–1478. 10.1128/MCB.19.2.1470. 9891080 PMC116075

[R2] Cao H, Bowling SA, Gordon AS, and Dong X (1994). Characterization of an Arabidopsis Mutant That Is Nonresponsive to Inducers of Systemic Acquired Resistance. Plant Cell 6:1583–1592. 10.1105/tpc.6.11.1583. 12244227 PMC160545

[R3] Chen J, Mohan R, Zhang Y, Li M, Chen H, Palmer IA, Chang M, Qi G, Spoel SH, Mengiste T, (2019). NPR1 promotes its own and target gene expression in plant defense by recruiting CDK8. Plant Physiol. 181:289–304. 10.1104/pp.19.00124. 31110139 PMC6716257

[R4] Clough SJ, and Bent AF (1998). Floral dip: a simplified method for Agrobacterium-mediated transformation of Arabidopsis thaliana. Plant J. 16:735–743. 10.1046/j.1365-313x.1998.00343.x. 10069079

[R5] Delannoy E, Batardiere B, Pateyron S, Soubigou-Taconnat L, Chiquet J, Colcombet J, and Lang J (2023). Cell specialization and coordination in Arabidopsis leaves upon pathogenic attack revealed by scRNA-seq. Plant Commun. 4:100676. 10.1016/j.xplc.2023.100676. 37644724 PMC10504604

[R6] Despres C, DeLong C, Glaze S, Liu E, and Fobert PR (2000). The Arabidopsis NPR1/NIM1 protein enhances the DNA binding activity of a subgroup of the TGA family of bZIP transcription factors. Plant Cell 12:279–290. 10662863 PMC139764

[R7] Ding Y, Sun T, Ao K, Peng Y, Zhang Y, Li X, and Zhang Y (2018). Opposite roles of salicylic acid receptors NPR1 and NPR3/NPR4 in transcriptional regulation of plant immunity. Cell 173:1454–1467.e15. 10.1016/j.cell.2018.03.044. 29656896

[R8] Dobin A, Davis CA, Schlesinger F, Drenkow J, Zaleski C, Jha S, Batut P, Chaisson M, and Gingeras TR (2013). STAR: ultrafast universal RNA-seq aligner. Bioinformatics 29:15–21. 10.1093/bioinformatics/bts635. 23104886 PMC3530905

[R9] Du Y, Zhao J, Chen T, Liu Q, Zhang H, Wang Y, Hong Y, Xiao F, Zhang L, Shen Q, (2013). Type I J-domain NbMIP1 proteins are required for both Tobacco mosaic virus infection and plant innate immunity. PLoS Pathog. 9:e1003659. 10.1371/journal.ppat.1003659. 24098120 PMC3789785

[R10] Fu ZQ, Yan S, Saleh A, Wang W, Ruble J, Oka N, Mohan R, Spoel SH, Tada Y, Zheng N, (2012). NPR3 and NPR4 are receptors for the immune signal salicylic acid in plants. Nature 486:228–232. 10.1038/nature11162. 22699612 PMC3376392

[R11] Heinz S, Benner C, Spann N, Bertolino E, Lin YC, Laslo P, Cheng JX, Murre C, Singh H, and Glass CK (2010). Simple combinations of lineage-determining transcription factors prime cis-regulatory elements required for macrophage and B cell identities. Mol. Cell 38:576–589. 10.1016/j.molcel.2010.05.004. 20513432 PMC2898526

[R12] Huang P, Dong Z, Guo P, Zhang X, Qiu Y, Li B, Wang Y, and Guo H (2020). Salicylic Acid Suppresses Apical Hook Formation via NPR1-Mediated Repression of EIN3 and EIL1 in Arabidopsis. Plant Cell 32:612–629. 10.1105/tpc.19.00658. 31888966 PMC7054027

[R13] Huang S, Zhu S, Kumar P, and MacMicking JD (2021). A phase-separated nuclear GBPL circuit controls immunity in plants. Nature 594:424–429. 10.1038/s41586-021-03572-6. 34040255 PMC8478157

[R14] Jin H, Choi SM, Kang MJ, Yun SH, Kwon DJ, Noh YS, and Noh B (2018). Salicylic acid-induced transcriptional reprogramming by the HAC-NPR1-TGA histone acetyltransferase complex in Arabidopsis. Nucleic Acids Res. 46:11712–11725. 10.1093/nar/gky847. 30239885 PMC6294559

[R15] Kagey MH, Newman JJ, Bilodeau S, Zhan Y, Orlando DA, van Berkum NL, Ebmeier CC, Goossens J, Rahl PB, Levine SS, (2010). Mediator and cohesin connect gene expression and chromatin architecture. Nature 467:430–435. 10.1038/nature09380. 20720539 PMC2953795

[R16] Kalde M, Barth M, Somssich IE, and Lippok B (2003). Members of the Arabidopsis WRKY group III transcription factors are part of different plant defense signaling pathways. Mol. Plant Microbe Interact. 16:295–305. 10.1094/MPMI.2003.16.4.295. 12744458

[R17] Kim JH, Castroverde CDM, Huang S, Li C, Hilleary R, Seroka A, Sohrabi R, Medina-Yerena D, Huot B, Wang J, (2022). Increasing the resilience of plant immunity to a warming climate. Nature 607:339–344. 10.1038/s41586-022-04902-y. 35768511 PMC9279160

[R18] Kim TW, Park CH, Hsu CC, Kim YW, Ko YW, Zhang Z, Zhu JY, Hsiao YC, Branon T, Kaasik K, (2023). Mapping the signaling network of BIN2 kinase using TurboID-mediated biotin labeling and phosphoproteomics. Plant Cell 35:975–993. 10.1093/plcell/koad013. 36660928 PMC10015162

[R19] Kinkema M, Fan W, and Dong X (2000). Nuclear localization of NPR1 is required for activation of PR gene expression. Plant Cell 12:2339–2350. 10.1105/tpc.12.12.2339. 11148282 PMC102222

[R20] Koidl S, and Timmers HTM (2021). greenCUT&RUN: efficient genomic profiling of GFP-tagged transcription factors and chromatin regulators. Curr. Protoc. 1:e266. 10.1002/cpz1.266. 34644460

[R21] Kong AT, Leprevost FV, Avtonomov DM, Mellacheruvu D, and Nesvizhskii AI (2017). MSFragger: ultrafast and comprehensive peptide identification in mass spectrometry-based proteomics. Nat. Methods 14:513–520. 10.1038/nmeth.4256. 28394336 PMC5409104

[R22] Kumar S, Zavaliev R, Wu Q, Zhou Y, Cheng J, Dillard L, Powers J, Withers J, Zhao J, Guan Z, (2022). Structural basis of NPR1 in activating plant immunity. Nature 605:561–566. 10.1038/s41586-022-04699-w. 35545668 PMC9346951

[R23] Langmead B, and Salzberg SL (2012). Fast gapped-read alignment with Bowtie 2. Nat. Methods 9:357–359. 10.1038/nmeth.1923. 22388286 PMC3322381

[R24] Li C, Gu L, Gao L, Chen C, Wei CQ, Qiu Q, Chien CW, Wang S, Jiang L, Ai LF, (2016). Concerted genomic targeting of H3K27 demethylase REF6 and chromatin-remodeling ATPase BRM in Arabidopsis. Nat. Genet. 48:687–693. 10.1038/ng.3555. 27111034 PMC5134324

[R25] Li H, Handsaker B, Wysoker A, Fennell T, Ruan J, Homer N, Marth G, Abecasis G, and Durbin R; 1000 Genome Project Data Processing Subgroup (2009). The Sequence Alignment/Map format and SAMtools. Bioinformatics 25:2078–2079. 10.1093/bioinformatics/btp352. 19505943 PMC2723002

[R26] Li J, Zhong R, and Palva ET (2017). WRKY70 and its homolog WRKY54 negatively modulate the cell wall-associated defenses to necrotrophic pathogens in Arabidopsis. PLoS One 12:e0183731. 10.1371/journal.pone.0183731. 28837631 PMC5570282

[R27] Li M, Chen H, Chen J, Chang M, Palmer IA, Gassmann W, Liu F, and Fu ZQ (2018). TCP Transcription Factors Interact With NPR1 and Contribute Redundantly to Systemic Acquired Resistance. Front. Plant Sci. 9:1153. 10.3389/fpls.2018.01153. 30154809 PMC6102491

[R28] Liu X, Sun Y, Korner CJ, Du X, Vollmer ME, and Pajerowska-Mukhtar KM (2015). Bacterial Leaf Infiltration Assay for Fine Characterization of Plant Defense Responses using the Arabidopsis thaliana-Pseudomonas syringae Pathosystem. J. Vis. Exp. e53364. 10.3791/53364. PMC4692633 26485301

[R29] Love MI, Huber W, and Anders S (2014). Moderated estimation of fold change and dispersion for RNA-seq data with DESeq2. Genome Biol. 15:550. 10.1186/s13059-014-0550-8. 25516281 PMC4302049

[R30] Mair A, Xu SL, Branon TC, Ting AY, and Bergmann DC (2019). Proximity labeling of protein complexes and cell-type-specific organellar proteomes in Arabidopsis enabled by TurboID. Elife 8:e47864. 10.7554/eLife.47864. 31535972 PMC6791687

[R31] Malamy J, Carr JP, Klessig DF, and Raskin I (1990). Salicylic Acid: a likely endogenous signal in the resistance response of tobacco to viral infection. Science 250:1002–1004. 10.1126/science.250.4983.1002. 17746925

[R32] Maleck K, Levine A, Eulgem T, Morgan A, Schmid J, Lawton KA, Dangl JL, and Dietrich RA (2000). The transcriptome of Arabidopsis thaliana during systemic acquired resistance. Nat. Genet. 26:403–410. 10.1038/82521. 11101835

[R33] Mann R, and Notani D (2023). Transcription factor condensates and signaling driven transcription. Nucleus 14:2205758. 10.1080/19491034.2023.2205758. 37129580 PMC10155639

[R34] Martin M (2011). Cutadapt removes adapter sequences from high-throughput sequencing reads. EMBnet. J. 17:10–12. 10.14806/ej.17.1.200.

[R35] Meers MP, Janssens DH, and Henikoff S (2019a). Pioneer factor-nucleosome binding events during differentiation are motif encoded. Mol. Cell 75:562–575.e5. 10.1016/j.molcel.2019.05.025. 31253573 PMC6697550

[R36] Meers MP, Bryson TD, Henikoff JG, and Henikoff S (2019b). Improved CUT&RUN chromatin profiling tools. Elife 8:e46314. 10.7554/eLife.46314. 31232687 PMC6598765

[R37] Metraux JP, Signer H, Ryals J, Ward E, Wyss-Benz M, Gaudin J, Raschdorf K, Schmid E, Blum W, and Inverardi B (1990). Increase in salicylic Acid at the onset of systemic acquired resistance in cucumber. Science 250:1004–1006. 10.1126/science.250.4983.1004. 17746926

[R38] Mi H, Muruganujan A, Casagrande JT, and Thomas PD (2013). Large-scale gene function analysis with the PANTHER classification system. Nat. Protoc. 8:1551–1566. 10.1038/nprot.2013.092. 23868073 PMC6519453

[R39] Moll P, Ante M, Seitz A, and Reda T (2014). QuantSeq 3’ mRNA sequencing for RNA quantification. Nat. Methods 11, i–iii. 10.1038/nmeth.f.376.

[R40] Mori S, Oya S, Takahashi M, Takashima K, Inagaki S, and Kakutani T (2023). Cotranscriptional demethylation induces global loss of H3K4me2 from active genes in Arabidopsis. EMBO J. 42:e113798. 10.15252/embj.2023113798. 37849386 PMC10690457

[R41] Nizamuddin S, Koidl S, Bhuiyan T, Werner TV, Biniossek ML, Bonvin AMJJ, Lassmann S, and Timmers H (2021). Integrating quantitative proteomics with accurate genome profiling of transcription factors by greenCUT&RUN. Nucleic Acids Res. 49:e49. 10.1093/nar/gkab038. 33524153 PMC8136828

[R42] Nobori T, Monell A, Lee TA, Zhou J, Nery J, and Ecker JR (2023). Time-resolved single-cell and spatial gene regulatory atlas of plants under pathogen attack. Preprint at bioRxiv. 10.1101/2023.04.10.536170.

[R43] Nomoto M, Skelly MJ, Itaya T, Mori T, Suzuki T, Matsushita T, Tokizawa M, Kuwata K, Mori H, Yamamoto YY, (2021). Suppression of MYC transcription activators by the immune cofactor NPR1 fine-tunes plant immune responses. Cell Rep. 37:110125. 10.1016/j.celrep.2021.110125. 34910911

[R44] O’Malley RC, Huang SSC, Song L, Lewsey MG, Bartlett A, Nery JR, Galli M, Gallavotti A, and Ecker JR (2016). Cistrome and Epicistrome Features Shape the Regulatory DNA Landscape. Cell 165:1280–1292. 10.1016/j.cell.2016.04.038. 27203113 PMC4907330

[R45] Olate E, Jiménez-Gómez JM, Holuigue L, and Salinas J (2018). NPR1 mediates a novel regulatory pathway in cold acclimation by interacting with HSFA1 factors. Nat. Plants 4:811–823. 10.1038/s41477-018-0254-2. 30250280

[R46] Perez-Riverol Y, Bai J, Bandla C, García-Seisdedos D, Hewapathirana S, Kamatchinathan S, Kundu DJ, Prakash A, Frericks-Zipper A, Eisenacher M, (2022). The PRIDE database resources in 2022: a hub for mass spectrometry-based proteomics evidences. Nucleic Acids Res. 50:D543–D552. 10.1093/nar/gkab1038. 34723319 PMC8728295

[R47] Ramirez F, Dundar F, Diehl S, Gruning BA, and Manke T (2014). deepTools: a flexible platform for exploring deep-sequencing data. Nucleic Acids Res. 42:W187–W191. 10.1093/nar/gku365. 24799436 PMC4086134

[R48] Rio DC, Ares M Jr., Hannon GJ, and Nilsen TW (2010). Purification of RNA using TRIzol (TRI reagent). Cold Spring Harb. Protoc. 2010:pdb.prot5439. 10.1101/pdb.prot5439. 20516177

[R49] Robinson JT, Thorvaldsdóttir H, Winckler W, Guttman M, Lander ES, Getz G, and Mesirov JP (2011). Integrative genomics viewer. Nat. Biotechnol. 29:24–26. 10.1038/nbt.1754. 21221095 PMC3346182

[R50] Saleh A, Withers J, Mohan R, Marqués J, Gu Y, Yan S, Zavaliev R, Nomoto M, Tada Y, and Dong X (2015). Posttranslational modifications of the master transcriptional regulator NPR1 enable dynamic but tight control of plant immune responses. Cell Host Microbe 18:169–182. 10.1016/j.chom.2015.07.005. 26269953 PMC4537515

[R51] Schneider CA, Rasband WS, and Eliceiri KW (2012). NIH Image to ImageJ: 25 years of image analysis. Nat. Methods 9:671–675. 10.1038/nmeth.2089. 22930834 PMC5554542

[R52] Seo SY, Wi SJ, and Park KY (2020). Functional switching of NPR1 between chloroplast and nucleus for adaptive response to salt stress. Sci. Rep. 10:4339. 10.1038/s41598-020-61379-3. 32152424 PMC7062895

[R53] Shannon P, Markiel A, Ozier O, Baliga NS, Wang JT, Ramage D, Amin N, Schwikowski B, and Ideker T (2003). Cytoscape: a software environment for integrated models of biomolecular interaction networks. Genome Res. 13:2498–2504. 10.1101/gr.1239303. 14597658 PMC403769

[R54] Singh M, Bag SK, Bhardwaj A, Ranjan A, Mantri S, Nigam D, Sharma YK, and Sawant SV (2015). Global nucleosome positioning regulates salicylic acid mediated transcription in Arabidopsis thaliana. BMC Plant Biol. 15:13. 10.1186/s12870-014-0404-2. 25604550 PMC4318435

[R55] Skene PJ, and Henikoff S (2017). An efficient targeted nuclease strategy for high-resolution mapping of DNA binding sites. Elife 6:e21856. 10.7554/eLife.21856. 28079019 PMC5310842

[R56] Szklarczyk D, Gable AL, Lyon D, Junge A, Wyder S, Huerta-Cepas J, Simonovic M, Doncheva NT, Morris JH, Bork P, (2019). STRING v11: protein-protein association networks with increased coverage, supporting functional discovery in genome-wide experimental datasets. Nucleic Acids Res. 47:D607–D613. 10.1093/nar/gky1131. 30476243 PMC6323986

[R57] Tang Y, Ho MI, Kang BH, and Gu Y (2022). GBPL3 localizes to the nuclear pore complex and functionally connects the nuclear basket with the nucleoskeleton in plants. PLoS Biol. 20:e3001831. 10.1371/journal.pbio.3001831. 36269771 PMC9629626

[R58] Tyanova S, Temu T, Sinitcyn P, Carlson A, Hein MY, Geiger T, Mann M, and Cox J (2016). The Perseus computational platform for comprehensive analysis of (prote)omics data. Nat. Methods 13:731–740. 10.1038/nmeth.3901. 27348712

[R59] Wang D, Amornsiripanitch N, and Dong X (2006). A genomic approach to identify regulatory nodes in the transcriptional network of systemic acquired resistance in plants. PLoS Pathog. 2:e123. 10.1371/journal.ppat.0020123. 17096590 PMC1635530

[R60] Wang W, Withers J, Li H, Zwack PJ, Rusnac DV, Shi H, Liu L, Yan S, Hinds TR, Guttman M, (2020). Structural basis of salicylic acid perception by Arabidopsis NPR proteins. Nature 586:311–316. 10.1038/s41586-020-2596-y. 32788727 PMC7554156

[R61] Weigel RR, Pfitzner UM, and Gatz C (2005). Interaction of NIMIN1 with NPR1 modulates PR gene expression in Arabidopsis. Plant Cell 17:1279–1291. 10.1105/tpc.104.027441. 15749762 PMC1088002

[R62] Wickham H (2016). ggplot2: Elegant Graphics for Data Analysis (New York: Springer-Verlag).

[R63] Wu Y, Zhang D, Chu JY, Boyle P, Wang Y, Brindle ID, De Luca V, and Desprós C (2012). The Arabidopsis NPR1 protein is a receptor for the plant defense hormone salicylic acid. Cell Rep. 1:639–647. 10.1016/j.celrep.2012.05.008. 22813739

[R64] Xu SL, Shrestha R, Karunadasa SS, and Xie PQ (2023). Proximity Labeling in Plants. Annu. Rev. Plant Biol. 74:285–312. 10.1146/annurev-arplant-070522-052132. 36854476 PMC10576617

[R65] Xu X, Chen C, Fan B, and Chen Z (2006). Physical and functional interactions between pathogen-induced Arabidopsis WRKY18, WRKY40, and WRKY60 transcription factors. Plant Cell 18:1310–1326. 10.1105/tpc.105.037523. 16603654 PMC1456877

[R66] Yun SH, Khan IU, Noh B, and Noh YS (2024). Genomic overview of INA-induced NPR1 targeting and transcriptional cascades in Arabidopsis. Nucleic Acids Res. 52:3572–3588. 10.1093/nar/gkae019. 38261978 PMC11039990

[R67] Zavaliev R, Mohan R, Chen T, and Dong X (2020). Formation of NPR1 condensates promotes cell survival during the plant immune response. Cell 182:1093–1108.e18. 10.1016/j.cell.2020.07.016. 32810437 PMC7484032

[R68] Zhang X, Yao J, Zhang Y, Sun Y, and Mou Z (2013). The Arabidopsis Mediator complex subunits MED14/SWP and MED16/SFR6/IEN1 differentially regulate defense gene expression in plant immune responses. Plant J. 75:484–497. 10.1111/tpj.12216. 23607369

[R69] Zhang Y, Fan W, Kinkema M, Li X, and Dong X (1999). Interaction of NPR1 with basic leucine zipper protein transcription factors that bind sequences required for salicylic acid induction of the PR-1 gene. Proc. Natl. Acad. Sci. USA 96:6523–6528. 10.1073/pnas.96.11.6523. 10339621 PMC26915

[R70] Zhang Y, Liu T, Meyer CA, Eeckhoute J, Johnson DS, Bernstein BE, Nusbaum C, Myers RM, Brown M, Li W, (2008). Model-based analysis of ChIP-Seq (MACS). Genome Biol. 9:R137. 10.1186/gb-2008-9-9-r137. 18798982 PMC2592715

[R71] Zheng XY, and Gehring M (2019). Low-input chromatin profiling in Arabidopsis endosperm using CUT&RUN. Plant Reprod. 32:63–75. 10.1007/s00497-018-00358-1. 30719569

[R72] Zhou JM, Trifa Y, Silva H, Pontier D, Lam E, Shah J, and Klessig DF (2000). NPR1 differentially interacts with members of the TGA/OBF family of transcription factors that bind an element of the PR-1 gene required for induction by salicylic acid. Mol. Plant Microbe Interact. 13:191–202. 10.1094/MPMI.2000.13.2.191. 10659709

[R73] Zhu J, Lolle S, Tang A, Guel B, Kvitko B, Cole B, and Coaker G (2023). Single-cell profiling of Arabidopsis leaves to Pseudomonas syringae infection. Cell Rep. 42:112676. 10.1016/j.celrep.2023.112676. 37342910 PMC10528479

[R74] Zhu W, Smith JW, and Huang CM (2010). Mass spectrometry-based label-free quantitative proteomics. J. Biomed. Biotechnol. 2010:840518. 10.1155/2010/840518. 19911078 PMC2775274

