# Supplementary material for: Next-generation mapping of the salicylic acid signaling hub and transcriptional cascade
Source: Mol Plant. Author manuscript; Available in PMC 2024 Nov 6. (PMC11540436; doi:10.1016/j.molp.2024.08.008)
Supplement: Powers et al., Supplemental information [file NIHMS2031245-supplement-Powers_et_al___Supplemental_information.pdf]

Molecular Plant, Volume *17*

Supplemental information

Next-generation mapping of the salicylic acid signaling hub and transcriptional cascade

Jordan Powers, Xing Zhang, Andres V. Reyes, Raul Zavaliev, Roni Ochakovski, Shou-Ling Xu, and Xinnian Dong

TITLE

Next-generation mapping of the salicylic acid signaling hub and transcriptional cascade

AUTHORS

Jordan Powers[1,2], Xing Zhang[1], Andres V. Reyes[3], Raul Zavaliev[4], Roni Ochakovski[1], Shou-Ling Xu[3], and Xinnian Dong[1,2*]

AFFILIATIONS

[1] Howard Hughes Medical Institute, Duke University, Durham, NC 27708, USA

[2]University Program in Genetics and Genomics, Duke University, Durham, NC 27708, USA

[3]Carnegie Institute for Science, Stanford University, Stanford, CA 94305, USA

[4]Department of Biology, Brookhaven National Laboratory, Upton, NY 11973, USA

**Correspondence: xdong@duke.edu*

RUNNING TITLE

Mapping the salicylic acid signal hub and cascade

Supplemental Figures, Figure Legends and Information

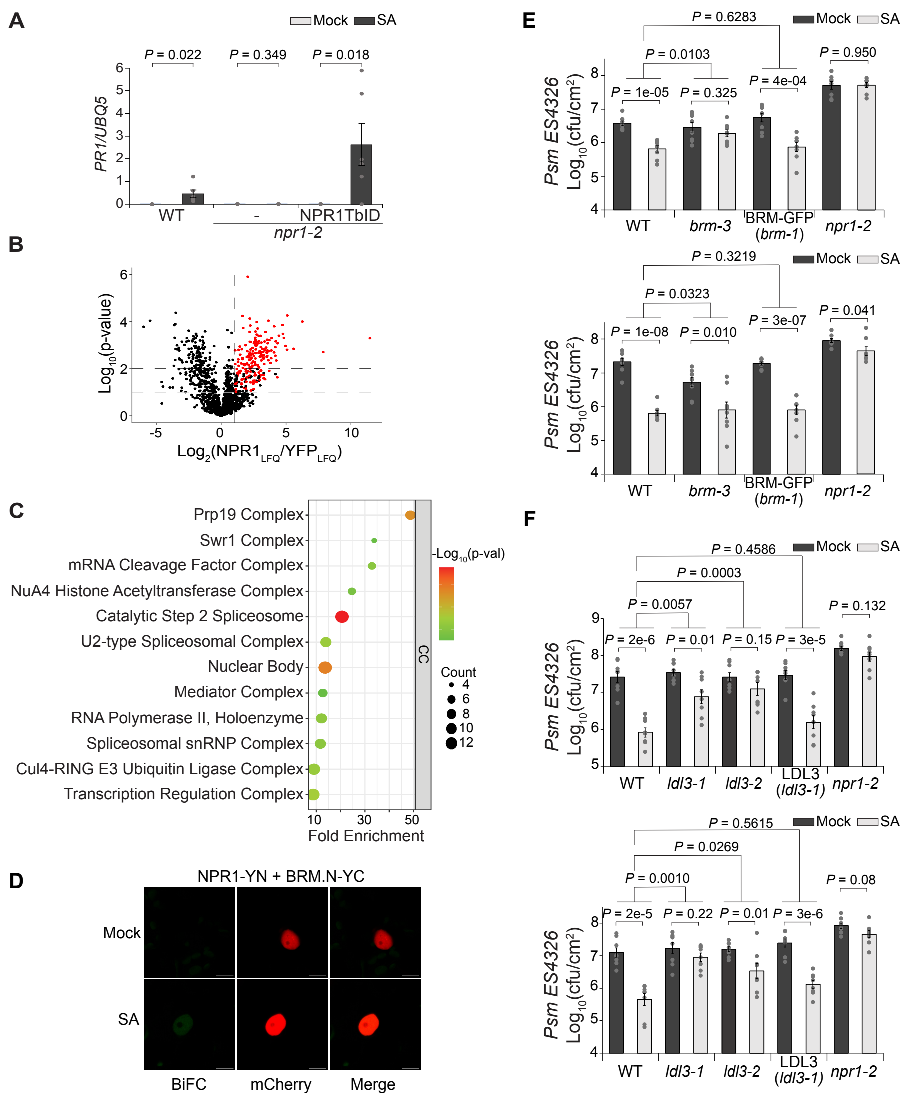

Supplemental Figure 1. NPR1-TbID is biologically active and interacts with splicing and transcriptional machineries in the nucleus upon SA induction. (A) *PR1* expression in WT, *npr1-2*, and *35S:NPR1-3xHA-TbID/npr1-2* (*NPR1-TbID*) complementation plants treated with H_2O (mock) or 1 mM SA for 24 h. (n = 6; error bars represent SEM, two-sided t-test was used to compare mock and 1 mM SA-treated samples). **(B)** NPR1 proximal proteins 4 h after treatment with 1 mM SA detected through TurboID biotin affinity purification followed by Label Free Quantification (LFQ) Mass Spectrometry processed under harsh conditions (see Methods). Red points represent proteins with a $NPR1_{LFQ}/YFP_{LFQ} \geq 2$ and p-

value < 0.1 in both mild and harsh washing conditions (see Methods) or p-value < 0.01 in at least one washing condition. **(C)** The enriched cellular components (CC) of the 234 NPR1 proximal proteins. **(D)** Proximity between NPR1 and BRM N-terminus. nYFP-fused NPR1 (NPR1-YN) and cYFP-fused BRM N-terminus (amino acids 1-952) (BRM.N-YC) were co-expressed with HTB1-mCherry in *N. benthamiana*. Plants were imaged after treatment with water (Mock) or 1 mM SA for 8 h. **(E and F)** Experimental replicates of bacterial colony-forming units (cfu) in WT, *brm-3*, *BRM-GFP* (*brm-1*) (**E**), *ldl3-1*, *ldl3-2*, *LDL3-FLAG* (**F**), and *npr1-2*. Plants were treated with H_2O (Mock) or 1 mM SA for 24 h before being inoculated with *Psm* ES4326 at $OD_{600\ nm}$ = 0.001. CFUs were measured 3 days post inoculation (n = 8; error bars represent SEM; two-sided t-test and two-way ANOVA were used for comparison within and between genotypes, respectively).

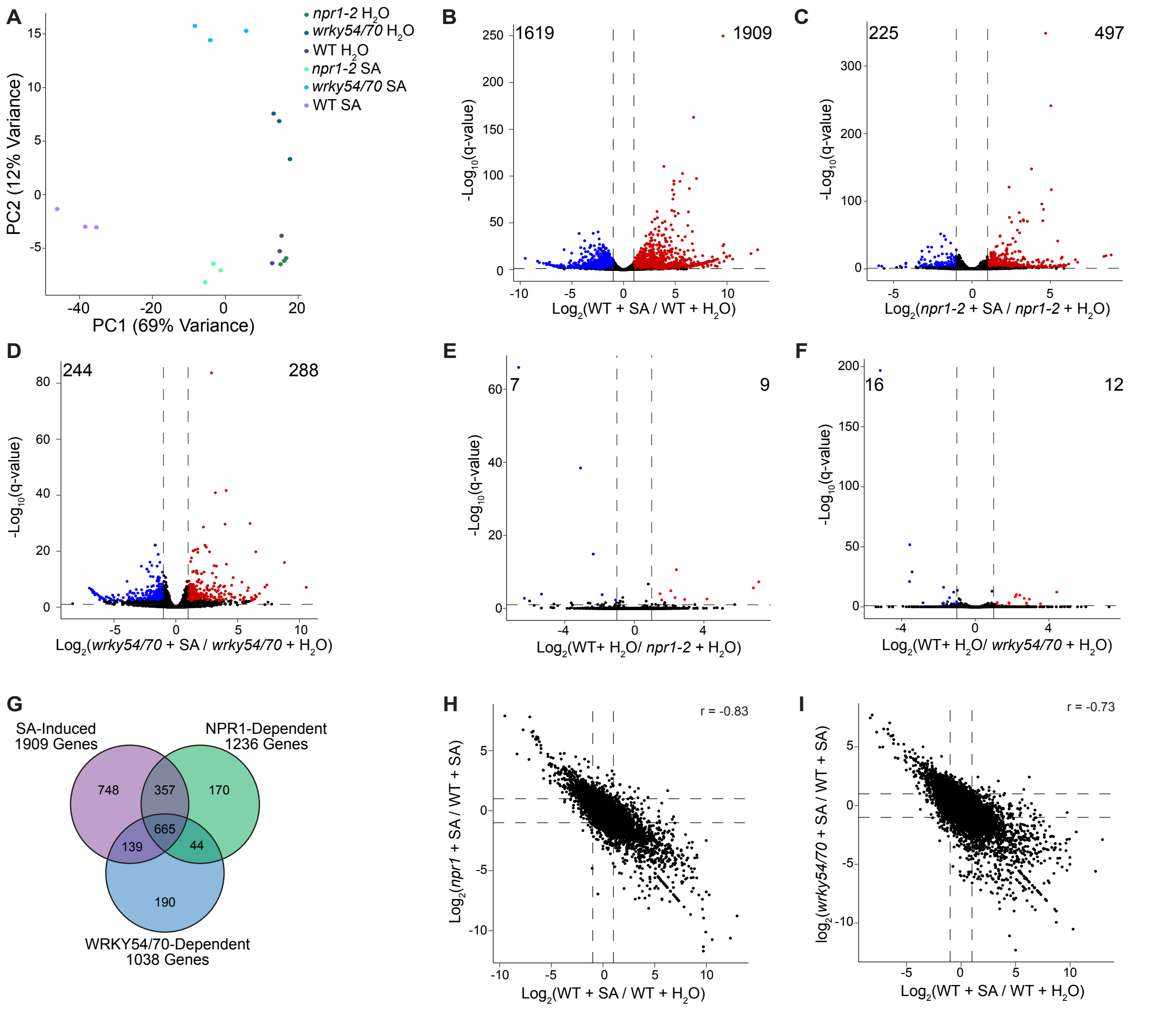

Supplemental Figure 2. SA-mediated transcriptional changes are partially dependent on NPR1 and/or WRKY54/70. (**A**) Principal component analysis (PCA) plot of WT, *npr1-2*, and *wrky54/70* QuantSeq samples treated with H_2O or 1 mM SA. (**B**) Volcano plot of SA-mediated transcriptional changes detected by QuantSeq. Colored points represent transcripts with (WT + SA) / (WT + H_2O) ≥ 2 (red) or ≤ -2 (blue) and an adjusted p-value (q-value) ≤ 0.1. (**C**) Volcano plot of NPR1-dependent transcriptional changes detected by QuantSeq. Colored points represent transcripts with (*npr1-2* + SA) / (*npr1-2* + H_2O) ≥ 2 (red) or ≤ -2 (blue) and an adjusted p-value (q-value) < 0.1. (**D**) Volcano plot of WRKY54/70-dependent transcriptional changes detected by QuantSeq. Colored points represent transcripts with (*wrky54/70* + SA) / (*wrky54/70* + H_2O) ≥ 2 (red) or < -2 (blue) and an adjusted p-value (q-value) ≤ 0.1. (**E**) Volcano plot of

basal differential transcripts in *npr1-2* compared to WT detected by QuantSeq. Colored points represent transcripts with (WT + H_2O) / (*npr1-2* + H_2O) ≥ 2 (red) or ≤ -2 (blue) and an adjusted p-value (q-value) ≤ 0.1. (**F**) Volcano plot of basal differential transcripts in *wrky54/70* compared to WT detected by QuantSeq. Colored points represent transcripts with (WT + H_2O) / (*wrky54/70* + H_2O) ≥ 2 (red) or ≤ -2 (blue) and an adjusted p-value (q-value) ≤ 0.1. (**G**) Venn diagram showing partial dependency of SA-mediated gene expression on NPR1 and/or WRKY54/70. (**H and I**) Relationship between NPR1 (**H**) or WRKY70 (**I**) and SA-mediated transcriptional reprogramming. r, Pearson correlation coefficient.

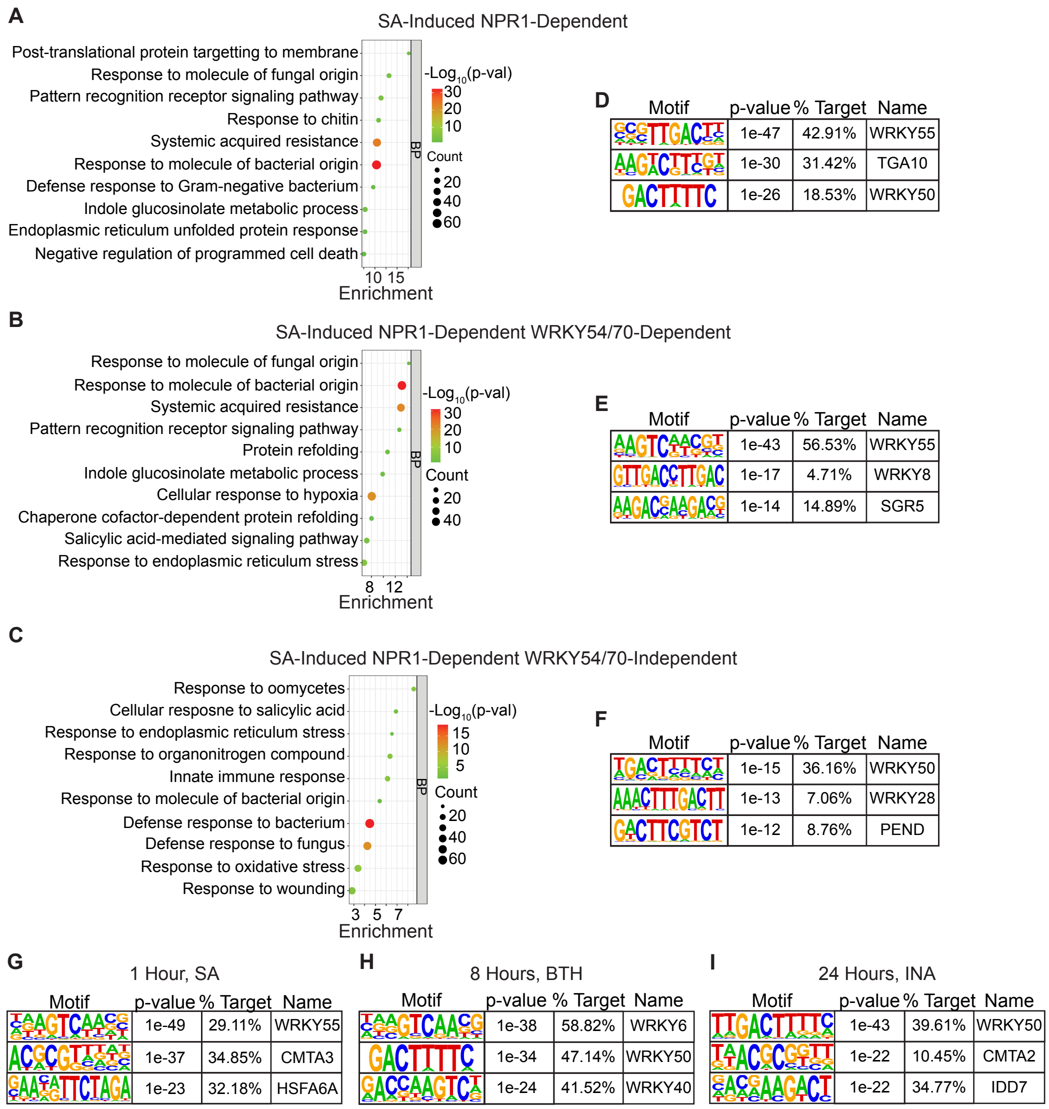

Supplemental Figure 3. NPR1- and/or WRKY54/70-dependent genes are enriched in defense-related biological processes and consistently enriched with the W-box motif. (A-C) Enriched biological processes (BP) in SA-induced NPR1-dependent genes (**A**), SA-induced NPR1-dependent WRKY54/70-dependent genes (**B**), and SA-induced NPR1-dependent and WRKY54/70-independent genes (**C**). **(D-F)** Motifs enriched from 1 kb upstream to 200 bp downstream of transcriptional start sites of the genes defined in **A-C**, respectively. **(G)** Enriched motifs of SA-induced genes 1 h after treatment determined by RNA-seq (Ding *et al.*, 2018). **(H)** Enriched motifs of the synthetic analog of SA, benzothiadiazole (BTH)-induced genes 8 h after treatment determined by microarray (Wang, Amornsiripanitch and Dong, 2006). **(I)**

Enriched motifs of the synthetic analog of SA, 2,6-dichloroisonicotinic acid (INA)-induced genes 24 h after treatment determined by RNA-seq (Jin *et al.*, 2018).

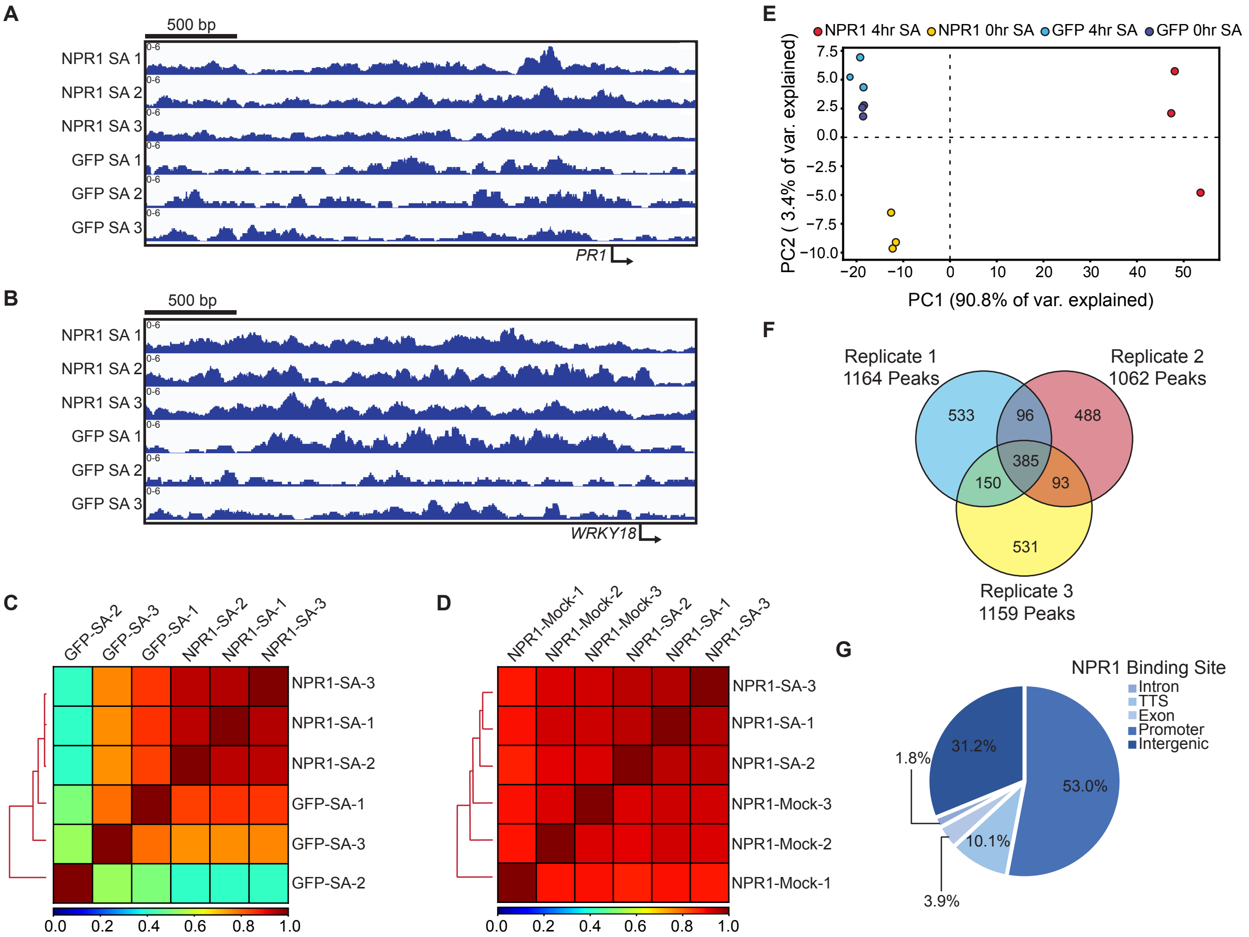

Supplemental Figure 4. CUT&RUN failed, while greenCUT&RUN succeeded, in detecting NPR1 binding to the chromatin in response to SA. (A and B) Integrative Genomics Viewer (IGV) of normalized NPR1-GFP (NPR1) and GFP reads at the promoters of known NPR1-target genes *PR1* (**A**) and *WRKY18* (**B**) 4 h after 1 mM SA treatment. Numbers represent 3 biological replicates for each genotype. **(C and D)** Pearson's correlation between NPR1 and GFP treated with SA (**C**), between NPR1-GFP treated with H_2O (mock) and SA (**D**). **(E)** Principal component analysis (PCA) of NPR1-GFP and GFP greenCUT&RUN data before and after treatment with 1 mM SA. **(F)** Venn diagram illustrating the reproducibility of greenCUT&RUN peaks among the three NPR1-GFP replicates 4 h after 1 mM SA treatment using GFP as the control. **(G)** Pie chart illustrating the locations of NPR1-GFP peaks in its target

genes defined as promoters (1 kb upstream to 1 bp upstream), intergenic (> 1 kb upstream), exon, intron, and transcriptional termination site (TTS).

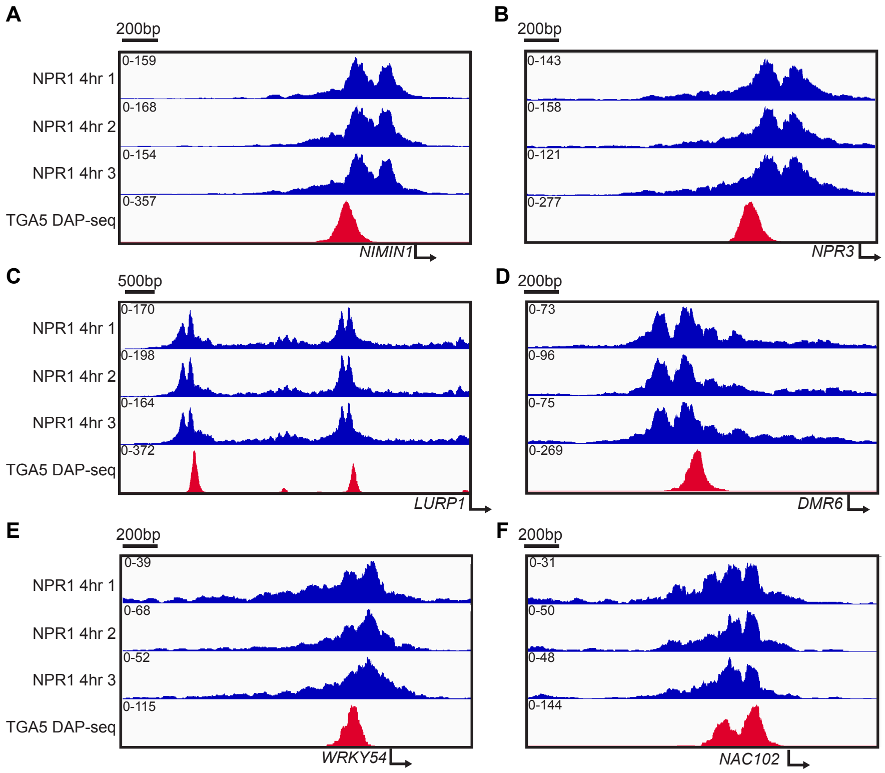

Supplemental Figure 5. TGA5 DAP-seq displays a close association of TGA5 to NPR1 binding loci. **(A-F)** Read Per Genomic Content (RPGC) normalized TGA5 (DAP-seq) (red) and NPR1 (greenCUT&RUN) (blue). RPGC at *NIMIN11* (**A**), *NPR3* (**B**), *LURP1* (**C**), *DMR6* (**D**), *WRKY54* (**E)**, and *NAC102* (**F**) promoter regions.

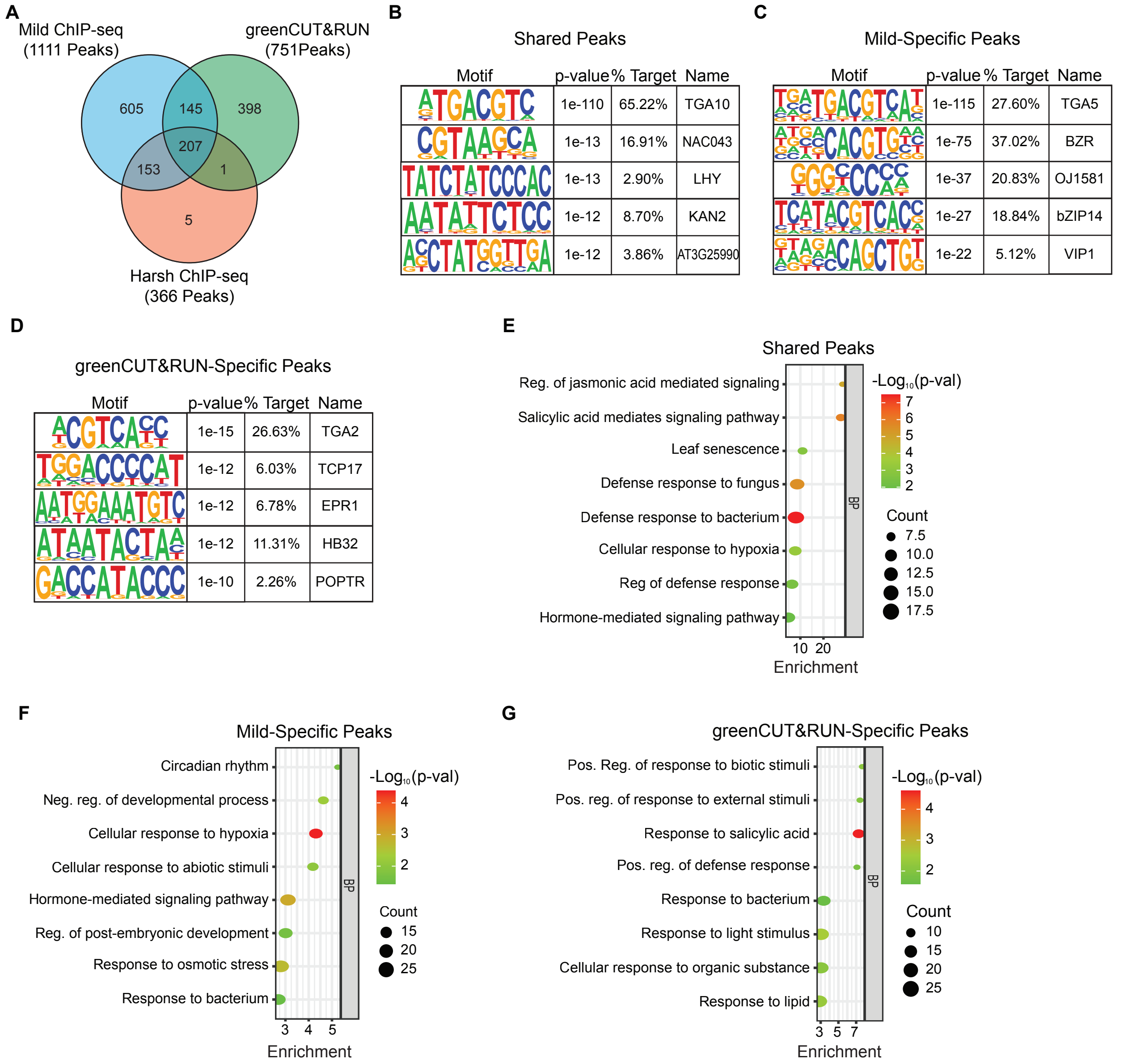

Supplemental Figure 6. NPR1 ChIP-seq detected shared and distinct peaks with those detected by greenCUT&RUN. (A) Venn diagram of NPR1 greenCUT&RUN peaks, NPR1 mild ChIP-seq peaks, and NPR1 harsh ChIP-seq peaks (Yun *et al.*, 2024). **(B - D)** Motifs enriched in the shared peaks (**B**), mild-specific peaks (**C**), and greenCUT&RUN-specific peaks **(D)**. **(E - G)** Enriched biological processes (BP) in NPR1 shared genes (**E**), mild-specific genes (**F**), and greenCUT&RUN-specific genes **(G)**.

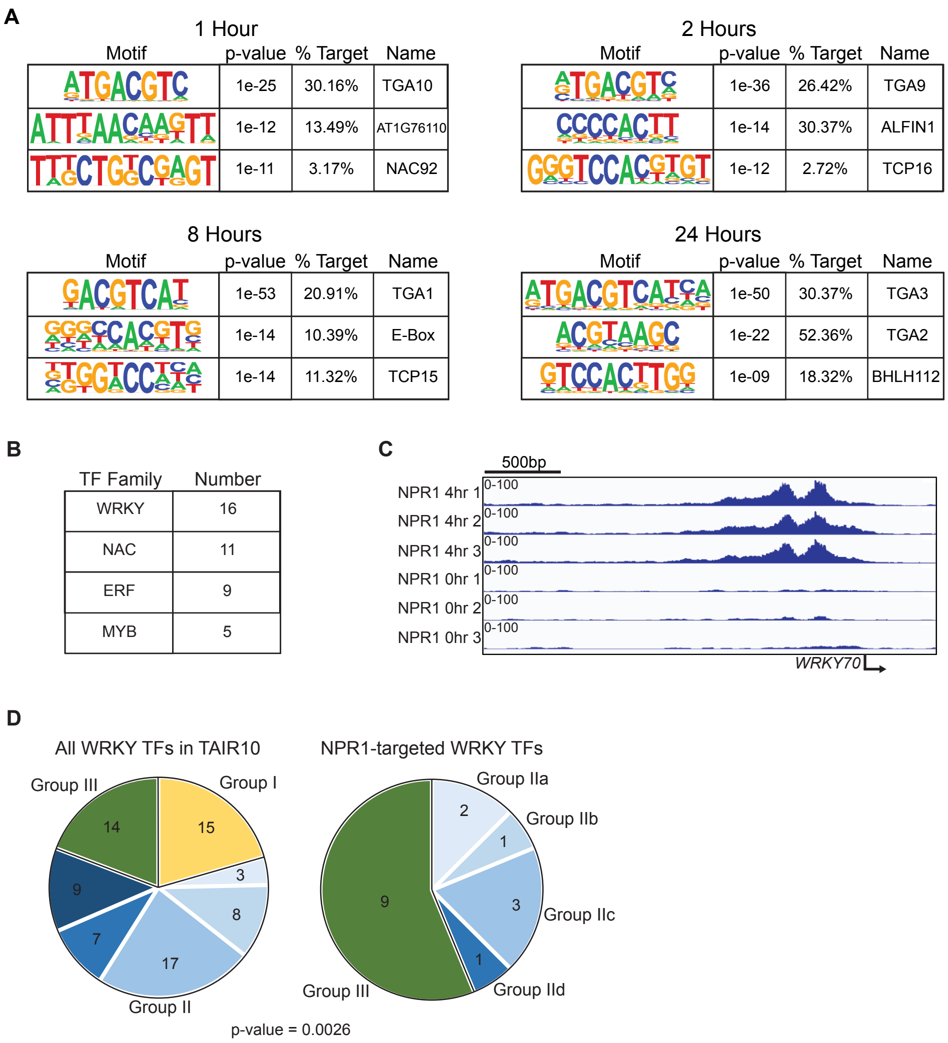

Supplemental Figure 7. NPR1 loci are continuously enriched with TGA binding element and NPR1 predominantly targets Group III WRKY TFs. (A) Motif enrichment at NPR1-GFP peaks detected 1, 2, 8, and 24 h after 1 mM SA treatment. **(B)** The most abundant TF families targeted by NPR1. **(C)** Integrative Genomics Viewer (IGV) of normalized NPR1 reads at the *WRKY70* promoter with and without SA treatment. Data from three biological replicates were used. **(D)** Pie charts of all *Arabidopsis WRKY* TF genes based on The Arabidopsis Information Resource 10 (TAIR10) compared to *WRKY* genes directly targeted by NPR1 (statistical significance determined by chi-square test).

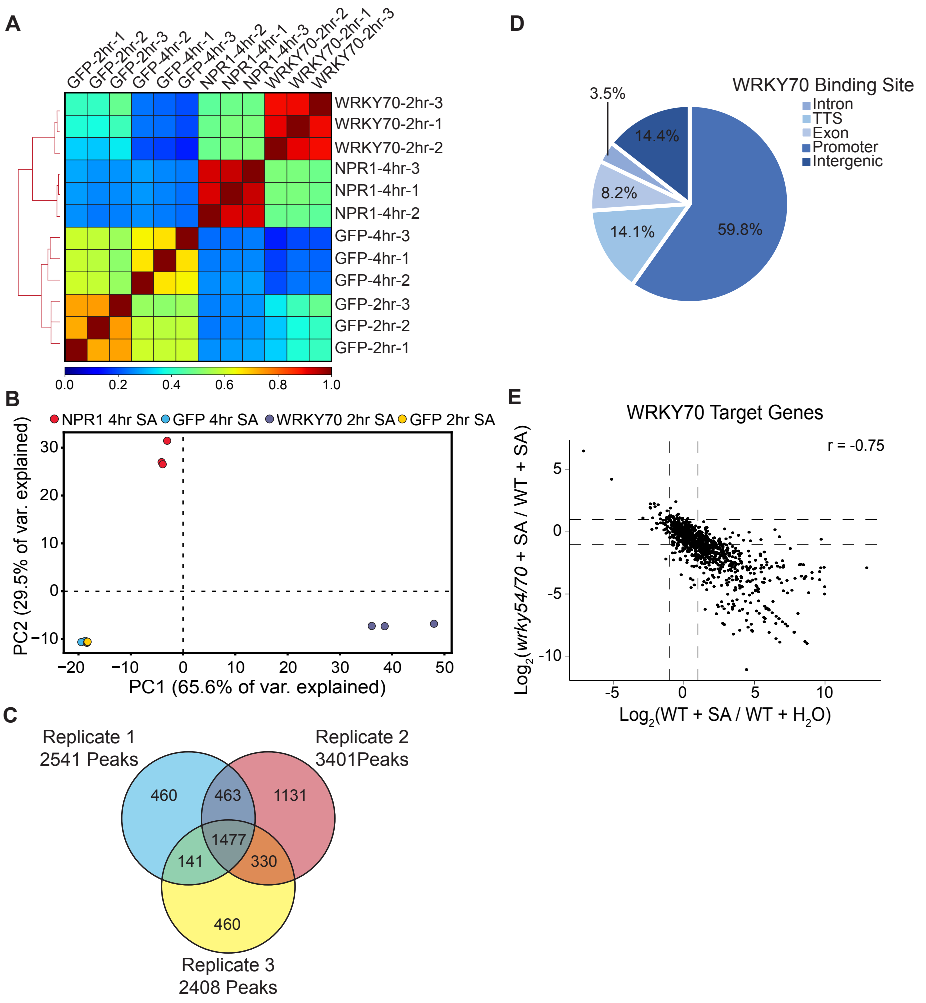

Supplemental Figure 8. greenCUT&RUN detection of WRKY70-GFP-binding at gene promoters upon SA induction. (A) Pearson correlation between WRKY70-GFP, NPR1-GFP, and GFP greenCUT&RUN data. **(B)** Principal component analysis (PCA) between WRKY70-GFP, NPR1-GFP, and GFP greenCUT&RUN data. **(C)** Venn diagram illustrating the reproducibility of greenCUT&RUN peaks among the three WRKY70-GFP replicates. **(D)** Pie chart illustrating the locations of WRKY70-GFP peaks in its target genes defined as promoters (1 kb upstream to 1 bp upstream), intergenic (> 1 kb upstream), exon, intron, and transcriptional termination site (TTS). **(E)** Correlation between SA-induced transcription and WRKY54/70-dependency at WRKY70-target genes. r, Pearson correlation coefficient.

Supplemental Information

Nuclei Isolation and greenCUT&RUN Buffers

Lysis Buffer (100 mL) – Nuclei Prep

Stock	Volume	Final Concentration
1 M Tris pH 7.5	2 mL	20 mM
80% glycerol	25 mL	20%
1 M KCl	2 mL	20 mM
0.5 M EDTA	0.4 mL	2 mM
1 M $MgCl_2$	0.2 5mL	2.5 mM
Sucrose solid	8.56 g Sucrose	8.56%
Protease Inhibitor		
dH_2O	Fill to 100mL	

NBRT (50 mL) – Nuclei Prep

Stock	Volume	Final Concentration
1 M Tris pH 7.5	1 mL	20 mM
80% Glycerol	12.5 mL	20%
1 M $MgCl_2$	0.125 mL	2.5 mM
10% Triton X-100	1 mL	0.2%
Protease Inhibitor		
dH_2O	Fill to 50mL	

Binding Buffer (50 mL) – Prep of ConA beads

Stock	Volume	Final Concentration
1 M HEPES-KOH pH7.5	1 mL	20 mM
1 M KCl	0.5 mL	10 mM
1 M $CaCl_2$	0.05 mL	1 mM
1 M $MnCl_2$ (Manganese Chloride)	0.5 mL	1 mM
dH_2O	fill to 50 mL	

Wash Buffer (200 mL) – greenCUT&RUN

Stock	Volume	Final Concentration
1 M HEPES-KOH pH 7.5	4 mL	20 mM
5 M NaCl	6 mL	150 mM
1.5 M spermidine	0.0667 mL (66.7 μL)	0.5 mM
Protease Inhibitor		
dH_2O	fill to 200 mL	

Wash Buffer + Ca^{2+} (5 mL) - greenCUT&RUN

Stock	Volume	Final Concentration
Wash Buffer	5 mL	1x
1 M $CaCl_2$	15 μL	3 mM

EDTA Buffer (50 mL) - greenCUT&RUN

Stock	Volume	Final Concentration
Wash Buffer	49.8 mL	1x
0.5 M EDTA	0.2 mL	2 mM EDTA

Stop Buffer (10 mL) - greenCUT&RUN

Stock	Volume	Final Concentration
5 M NaCl	680 µL	340 mM
0.5 M EDTA	400 µL	20 mM
0.5 M EGTA	200 µL	10 mM
20 mg/mL RNAse A	50 µL	100 µg/mL
15 mg/mL Glycogen (GlycoBlue)	33 µL	50 µg/mL
Spike-in DNA (if available)		
dH_2O	Fill to 10 mL	

Other reagents and special supplies needed

40 µm filter
70 µm filter
Phenol Chloroform Isoamyl Alcohol 25:24:1
Chloroform
100% Ethanol (Molecular Grade)
Phaselock or Maxtract tubes
DNA LoBind 1.7 mL tubes
DNA LoBind 96 well plate
enh-MNase (Addgene #166035)
Protease K
10% SDS
Concanavalin A beads (ConA Beads)
KAPA HyperPrep Kit
AMPure or KAPA cleanup beads

Nuclei Prep Protocol

Unless otherwise stated, always keep all buffers and tubes cold.

1. Flash Freeze Tissue, normally 6 leaves (leaves 4, 5, and 6 from two different 3-week-old plants) and grind into a fine powder in liquid nitrogen.
2. Add powder to a 50 mL conical tube on ice. Resuspend in 10 mL cold lysis buffer.
3. Pass the suspension through 70 μm and 40 μm filters.
4. Pellet nuclei by spinning in a pre-cooled centrifuge at 1,500 x g for 10 minutes at 4 °C.
5. Resuspend* the nuclei in 1 mL NBRT and transfer to a new 2 mL round bottom tube.
6. Spin again at 1,500 x g for 10 minutes at 4 °C.
    a. *ConA beads can be prepared during these spins.
7. Repeat washing (with NBRT) and spinning for a total of four washes.
    a. This step can be repeated until all chloroplasts are gone (no green).
    b. The color should be off-whiteish.
8. Resuspend the nuclei in 1 mL of CUT&RUN wash buffer.
9. Proceed to CUT&RUN protocol.

*Resuspend nuclei by gently pipetting up and down with a P-1000 μL using a cut tip.

*Nuclei can alternatively be isolated using FACS (PMID: 30719569).

greenCUT&RUN Protocol

1. Take 40 µL well-mixed ConA bead slurry per sample and move to a single tube.
   a. 10 µL of beads is used per sample. The ConA beads we use are a 25% slurry, so 40 µL is 10 µL of bead volume.
2. Place the tube on a magnetic rack and allow the solution to clear.
3. Remove the supernatant and wash twice by resuspending it in 1 mL binding buffer.
4. Resuspend beads in 10 µL binding buffer per sample (e.g.: resuspend all in 100 µL for 10 samples).
5. Add 10 µL of beads to 1 mL of nuclei in wash buffer and incubate on a rotator at room temperature for 10 minutes.
6. Place on a magnetic rack and allow the solution to clear. Once clear, remove the supernatant.
7. Add 1 mL of EDTA buffer and rotate tubes at room temperature for 10 minutes.
   a. This step removes any excess calcium that would prematurely activate enh-MNase.

Move to the cold room for steps 8-15.

8. Place tubes on a magnetic rack and allow the solution to clear. Remove liquid.
9. Wash beads in 1 mL of wash buffer.
10. Add 100 µL of wash buffer with enh-MNase. Rotate at 4 °C for 30 minutes.
    a. The final concentration of the enh-MNase should be 10 µg/mL.
11. Place tubes on a magnetic rack and wait for the solution to clear. Once clear, remove the supernatant.
12. Wash nuclei in 1 mL wash buffer twice.
13. On wet ice, resuspend nuclei in 150 µL of wash buffer with Ca^{2+}.
14. Incubate on wet ice for 30 minutes.
15. Add 100 µL of Stop buffer to the tubes.
16. Incubate tubes at 37 °C for 30 minutes.
17. Centrifuge tubes at 16,000 x g for 5 minutes at 4 °C, place on a magnetic rack, and transfer 200 µL of supernatant to a LoBind DNA tube.

DNA Isolation

1. Add 2 µL of 10% SDS and 1.5 µL proteinase K (20 mg/ml) to each tube and incubate at 50 °C for 1 hour.
2. Add equal volumes of 25:24:1 Phenol Chloroform Isoamyl Alcohol and vortex briefly.
3. Transfer the solution to a phase lock or Maxtract tube and centrifuge for 5 minutes at 16,000 x g at room temperature.
4. Add equal volumes of chloroform and invert tubes 10 times to mix.
5. Centrifuge for 5 minutes at 16,000 x g at room temperature.
6. Remove the top liquid and add it to a tube containing 3 µL of 2 mg/mL Glycoblue.
    a. 0.2 µL 15 mg/mL GlycoBlue and 2.8 µl water per sample.
7. Add 500 µL of 100% Molecular Biology Grade ethanol and chill in the -20 °C freezer overnight or longer.
8. Centrifuge for 10 minutes at 16,000 x g at 4 °C.
9. Pour off the liquid and drain on a paper towel.
10. Rinse the pellet in 1 mL of 100% molecular-grade ethanol.
11. Centrifuge for 3 minutes at 16,000 x g at 4 °C.
12. Pour off the liquid and drain on a paper towel. Continue to air dry the pellet.
13. Resuspend the pellet in 50 µL of dH_2O. Transfer to a LoBind 96 well plate and proceed to KAPA HyperPrep library prep.

Library Prep (Adapted from KAPA HyperPrep Kit)

https://sequencing.roche.com/us/en/products/group/kapa-hyperprep-kits.html

1. End Repair and A-Tailing
    a. Add 7 µL of End Repair & A Tailing Buffer and 3 µL of End Repair and A Tailing Enzyme Mix to each 50 µL sample.
        i. Make a master mix of the enzyme and buffer.
    b. Incubate at 20 °C for 30 minutes and 58 °C for 1 hour.
        i. This differs from the normal protocol, which used 65 °C for 30 minutes. For small DNA fragments, 65 °C is too high.

2. Adapter Ligation
    a. Add 5 µL of 1:100 dilute Illumina adapters, 5 µL of PCR grade water, 30 µL of Ligation Buffer, and 10 µL of DNA Ligase to each End Repaired and A-Tailed sample.
    b. Incubate at 20 °C for 30 minutes.

3. Post-ligation Clean-Up
    a. Add 100 µL of room temperature KAPA cleanup or AMPure beads to each sample and mix thoroughly.
        i. Depending on the expected fragment size the volume of beads can be increased or decreased, with more beads including smaller DNA fragments.
    b. Incubate the plate for 10 minutes at room temperature.
    c. Place the rack on a magnet and allow the solution to clear.
    d. Carefully remove the supernatant.
    e. Wash the beads twice in 200 µL of 80% ethanol.
        i. Keep the plate on the magnetic stand.
    f. Let the beads dry for 3 minutes.
    g. Resuspend the beads in 55 µL of dH_2O.
    h. Incubate at room temperature for 10 minutes.
    i. Place the rack on a magnet and allow the solution to clear.
    j. Move 50 µL of supernatant into a new well.
    k. Add 60 µL of room temperature KAPA cleanup or AMPure beads to each sample and mix thoroughly.
        i. Depending on the expected fragment size the volume of beads can be increased or decreased, with more beads including smaller DNA fragments.
    l. Repeat Steps **b – j**.
        i. This is different than the normal protocol. Because of how little DNA there is, the first clean-up does not size select well.

4. Double Size Selection
   a. Add 35 µL of KAPA cleanup or AMPure beads to each sample and mix thoroughly.
   b. Incubate at room temperature for 10 minutes.
   c. Place the rack on a magnet and allow the solution to clear.
   d. Carefully move the **<u>SUPERNATANT</u>** to a new well.
      i. The bead will currently bind the large DNA fragments (non-specific, sheered chromatin), while the small fragments (the fragments of interest) will be in the supernatant.
   e. Add 25 µL of KAPA cleanup or AMPure beads to each sample and mix thoroughly.
   f. Incubate at room temperature for 10 minutes.
   g. Place the rack on a magnet and allow the solution to clear.
   h. Remove the supernatant and wash the beads with 200 µL of 80% ethanol while keeping the plate on the magnetic rack.
   i. Allow the beads to dry for 3 minutes.
   j. Resuspend the beads in 25 µL of dH_2O.
   k. Incubate at room temperature for 10 minutes.
   l. Place the rack on a magnet and allow the solution to clear.
   m. Move 20 µL of supernatant into a new well.

5. Library Amplification
   a. Add 25 µL of HiFi HotStart ReadyMix (2X) and 5 µL of KAPA Library Amplification Mix (10X) to each 20 µL sample.
      i. Make a master mix of the ReadyMix and Library Amplification Mix.
      ii. Be very cautious with the Library Amplification Mix as the stock can easily overwhelm the sequencing run if introduced.
   b. Amplify as follows

Step	Temp	Duration	Cycles
Initial denaturation	98 °C	45 sec	1
Denaturation	98 °C	15 sec	X
Annealing	60 °C	10 sec	
Extension	72 °C	10 sec	
Final extension	72 °C	1 min	1
Hold	4 °C	∞	1

      i. X will vary depending on how much the library needs to be amplified. For TFs, 16 cycles. For non-DNA binding proteins, 18 was used. This can be modified and adjusted as needed.

ii. Annealing and extension are much shorter than the normal protocol. This is to further select for smaller fragments.

6. Post-amplification Cleanup
    a. Add 60 µL of room temperature KAPA cleanup or AMPure beads to each sample and mix thoroughly.
    b. Incubate the plate for 10 minutes at room temperature.
    c. Place the rack on a magnet and allow the solution to clear.
    d. Carefully remove the supernatant.
    e. Wash the beads twice in 200 µL of 80% ethanol.
        i. Keep the plate on the magnetic stand.
    f. Let the beads dry for 3 minutes.
    g. Resuspend the beads in 55 µL of dH_2O.
    h. Incubate at room temperature for 10 minutes.
    i. Place the rack on a magnet and allow the solution to clear.
    j. Move 50 µL of supernatant into a new well.
    k. Repeat steps a – f.
    l. Resuspend the beads in the final volume needed for sequencing + 3 µL.
        i. Normally 28 µL is used for eluting and 25 µL for QC and sequencing.
    m. Incubate at room temperature for 10 minutes.
    n. Place the rack on a magnet and allow the solution to clear.
    o. Move 25 µL of supernatant into a new well.
        i. Or the amount needed for QC and sequencing.
    p. Proceed to library QC using Qubit and Bioanalyzer.
    q. Send libraries for sequencing.
        i. Libraries are stable at 4 °C for 1 week of 3 months at -20 °C.
